# Global burden of female breast cancer and its association with socioeconomic development status, 1990–2044

**DOI:** 10.1002/cnr2.1827

**Published:** 2023-04-24

**Authors:** Jingya Zhang, Yongbo Lu, Ning Zhang, Zeru Yu, Haorao Li, Rongxin He, Ying Mao, Bin Zhu

**Affiliations:** ^1^ School of Public Policy and Administration Xi'an Jiaotong University Xi'an China; ^2^ Vanke School of Public Health Tsinghua University Beijing China; ^3^ School of Public Health and Emergency Management Southern University of Science and Technology Shenzhen Guangdong China

**Keywords:** female breast cancer, global, incidence, prediction, risk factor

## Abstract

**Background:**

Breast cancer is a widespread disease in women worldwide.

**Aim:**

We aimed to explore the global epidemiological trends of female breast cancer (FBC) between 1990 and 2044.

**Methods and Results:**

Disease burden, population, and socio‐demographic index (SDI) data were obtained from the Global Health Data Exchange (GHDx) database. We analyzed temporal trends, age differences, risk factors, and geographic patterns of FBC disease burden globally and explored the association between age‐standardized incidence rate (ASIR) of FBC and SDI. Bayesian age‐period‐cohort model was also performed to predict the changes in FBC incidence worldwide from 2020 to 2044. First, the global ASIR of FBC increased by 14.31% from 1990 to 2019 (95% Uncertainty Interval 4.75% to 23.98%). The death rate presented a falling trend. Second, alcohol use is the most‐highlighted risk factor for FBC in some high‐income regions such as Europe. A high fasting plasma glucose levels is the most prominent risk factor for FBC in Latin America and Africa. Third, the ASIR of the FBC increases with the SDI. Fourth, the incidence is expected to increase faster among women aged 35–60 years and fastest for those aged 50–54 years from 2020 to 2044. Countries with a high incidence of FBC that is expected to increase significantly include Barbados, Burkina Faso, Senegal, Monaco, Lebanon, Togo, and Uganda.

**Conclusion:**

The disease burden of FBC varies worldwide; the findings suggest attaching importance to the control of middle and low‐middle SDI regions. Public health as well as cancer prevention experts should pay more attention to regions and populations at an increased risk of developing FBC, focusing on their prevention and rehabilitation while conducting further epidemiological studies to investigate the risk factors of their increase.

## 1 BACKGROUND

The incidence of breast cancer is increasing worldwide, with 4.4 million cases predicted by 2070.^1^ Women constitute the majority of breast cancer patients, and female breast cancer (FBC) remains widespread among women in most regions.^2^ FBC accounts for roughly 24.5% of all cancer cases and for 15.5% of cancer deaths in women, leading in most countries in terms of incidence and death rates in 2020.^3^ There are considerable variations in the morbidity, death, and survival rates of FBC between regions.^4^ Age‐standardized incidence rate (ASIR) ranged from 112.3/100000 in Belgium to 35.8/100000 in Iran, while age‐standardized death rate (ASDR) ranged from a high of 41.0 per 100 000 in Fiji to a low of 6.4 per 100 000 in Korea.^5^ Therefore, FBC is a public health issue that warrants attention.

FBC risk factors are multifaceted and include both uncontrollable (age,^6^ family history,^7^ race^8^) and controllable (BMI,^6^ smoking,^6^ alcohol consumption,^9^ diabetes,^10^ timing of first delivery,^11^ breastfeeding,^6^ organic solvent occupational exposure,^12^ and electromagnetic occupational exposure^13^) factors. Meanwhile, COVID‐19 had a significant impact on FBC screening, breast surgery, and genetic counseling,^14^ with a general decrease in the number of patients accessing prevention, screening, diagnosis, and treatment during the outbreak.

Local management of FBC, adjuvant systemic therapy, and treatment of patients with advanced disease have evolved in recent years^15^ toward identifying more conservative ways to treat the cancer and provide the best quality of life for patients.^16^ However, no valid vaccine has yet been produced to protect against FBC.^17^ Furthermore, extended and more active FBC therapies have raised the prevalence of long‐term survival.^18^ Therefore, it is necessary to accurately predict the risk of FBC and develop individualized strategies to identify better ways to prevent the disease.

Most previous studies were based on all breast cancer patients,^19^ and the prediction is localized.^20^ This study focuses on FBC, with the prediction covering all countries. We analyzed the temporal trends, age differences, risk factors, and geographic patterns of FBC disease burden worldwide and examined the association between ASIR and socio‐demographic index (SDI). In addition, we made age‐specific and country‐specific projections of changes in FBC incidence worldwide from 2020 to 2044 to better identify populations at increased risk of FBC. On this basis, trends worthy of public health and cancer prevention experts' close attention were highlighted.

## 2 METHODS

### 2.1 Data source

Disease burden data were obtained for FBC In the “Global Burden of Disease Study 2019” ((GBD)2019) using the Global Health Data Exchange (GHDx) query tool (http://ghdx.healthdata.org/gbd-results-tool). The incidence, death, Disability adjusted life year (DALY), and percentage change in 1990–2019 were extracted by age, sex, region, country, and risk factors. According to the socio‐demographic situation, regions and countries were classified into five levels. According to geographical features, there are 21 Global Burden of Disease (GBD) regions. The data covered 204 countries/regions worldwide. The calculation of estimates in the GBD 2019 database was based on those described in previous studies.^21^, ^22^, ^23^

Population estimates for 5‐year and custom age groups were gained from the World Population Prospects 2022 (https://population.un.org/wpp/Download/Standard/Population/) from 1990 to 2044.

SDI data, a combined indicator of income, education, and fertility,^24^ were also gained using the GHDx query tool (https://ghdx.healthdata.org/record/ihme-data/gbd-2019-socio-demographic-index-sdi-1950-2019), according to which SDI data were collected for 21 GBD regions worldwide from 1990 to 2019.

This is original study and all the datasets were obtained from publicly accessible databases. No ethics approval was required. This exemption is in accordance with the Chinese ethical review policy guidelines (http://www.gov.cn/zhengce/zhengceku/2023-02/28/content_5743658.htm) (National Health Science and Education Development [2023] No. 4, clause 1 of section 32).

### 2.2 Data analysis

We described the trend of an interval or a whole period using the average annual percentage change (AAPC) and its 95% Uncertainty Interval (UI). This metric is derived from the Joinpoint regression analysis, which has been widely used to analyze cancer mortality and incidence data.^25^ In addition, we determined the annual percentage change in each identified trend of FBC rates using the calendar year as a regression variable. The AAPC throughout the considered period was also calculated. Based on a Poisson regression model, the positions of joinpoints and regression coefficients were estimated, while the optimal number of joinpoints was selected by means of a permutation test. Each *P*‐value was calculated using the Monte Carlo methods, and the overall asymptotic significance level was maintained through a Bonferroni correction.^26^ A *P*‐value of less than 0.05 was considered statistically significant. If the lower UI of AAPC is above 0, it reveals an uphill tendency of the indicator, and if the upper UI of AAPC is below 0, it indicates a downward trend of the indicator. Additionally, if the confidence interval contains 0, it indicates that the trend of change is not statistically significant.^27^

We obtained 30 years of data for 22 regions, with a total of 660 sets, based on which we explored the relationship between the ASIR and SDI of FBC. As this relationship is difficult to transform into a linear model with a simple function, a polynomial regression was performed. The general form of the polynomial is: $y=p_{0}x^{n}+p_{1}x^{n-1}+p_{2}x^{n-2}+p_{3}x^{n-3}+\ldots +p_{n}$. The purpose of the polynomial fit is to find a set of $p_{0}$, $p_{1}$, …$p_{n}$, so that the fitted equations match the actual sample data as closely as possible. The significance level was set at *P* < .05. The adjusted *R*‐squared was chosen to judge the fit of the model, which represents the proportion of the variance of y by the fitted values. For a series of true values ($y_{i}$) and fitted values (${\hat{y}}_{i}$), *R*‐squared is defined as $R^{2}=1-\frac{\sum_{i}{\left({\hat{y}}_{i}-y_{i}\right)}^{2}}{\sum_{i}{\left(y_{i}-\bar{y}\right)}^{2}}$. It takes a value between 0 and 1, with a value closer to 1 representing a better fit.^28^ We also examined the temporal trends in ASIR for the five SDI regions during 1990–2019.

We performed Bayesian age‐period‐cohort (APC) analyses for incidence prediction, which shows better coverage and precision than other prediction methods as it involves no parametric assumptions.^29^, ^30^ The APC model is based on Poisson distribution, which decomposes the target analysis variables from three dimensions of age, period, and cohort, allowing better reporting of the risk of disease onset. However, the linear relationship between these three components makes the complete model unidentifiable. Bayesian APC assumes that close time effects that are similar are attributed to priors' probability distributions. It is a hierarchical model that incorporates uncertainty about hyper parameters and avoids difficulties arising from the identifiability problem by applying mildly informative prior distributions. More information about Bayesian age‐period‐cohort analysis is shown in supplemental file 1.

For the global projections by age group, people were initially grouped into 14 groups (25–29,30–34,35–39,40–44,45–49,50–54, 55–59, 60–64, 65–69, 70–74, 75–79, 80–84, 85–89, and 90–94) according to their age. Second, the same five‐year interval was used to separate the period into six groups for 1990–2019. Finally, the incidence of FBC at 25–94 years of age was predicted globally for 2020–2044. In the country‐level forecast, similarly, age was to sorted into 18 groups (0–15, 15–19, 20–24, 25–29, 30–34, 35–39, 40–44, 45–49, 50–54, 55–59, 60–64, 65–69, 70–74, 75–79, 80–84, 85–89, 90–94, 95+). Second, we split the period into six groups. Based on these data, the age‐specific incidence was calculated. We also applied the United Nations' 2020 female demographic structure for standardization to obtain the ASIR for each country for 2020–2044.We modified Bayesian APC with the bamp and Nordpred packages in R (version 4.1.12) to foresee the incidence in 2020–2044. The maps were drawn using ArcMap 10.8. Polynomial fitting and figure plotting were performed using OriginPro (version 2020b).

## 3 RESULTS

### 3.1 Changes in female breast cancer burden worldwide from 1990 to 2019

Table 1 shows some disease burden indicators for FBC in each SDI and GBD region between 1990 and 2019. In 2019, the global ASIR of FBC was 45.8568 per 100 000 (41.9079 to 49.7581 per 100 000), with a 14.31% increase in percentage change from 1990 to 2019 (95% UI 4.75% to 23.98%). The global ASDR for FBC was 15.8838 per 100 000 (14.6557 to 17.0711 per 100 000), with a 10.54% decrease in percentage change between 1990 and 2019 (95% UI 4.23% to 17.33%). The global age‐standardized DALY rate for FBC was 473.8254 per 100 000 (437.2981 to 510.5076 per 100 000), with a decrease of 9.73% between 1990 and 2019 (95% UI 2.97% to 17.12%). Years of life lost (YLL) contributed most of the values of DALY, showing a downward trend from 1990 to 2019. However, years lived with disability (YLD) showed an upward trend. Data from the other regions are shown in supplemental file 2.

**TABLE 1:** ASID, ASDR, and age‐standardized DALY rate for FBC in 2019 and percentage change of age‐standardized rates globally

	Rate (Per 100 000)			Percentage change in age‐standardized rates, 1990–2019		
	Value	Upper	Lower	Value	Upper	Lower
**ASIR**	45.8568	49.7581	41.9079	0.1431	0.2398	0.0475
**ASDR**	15.8838	17.0711	14.6557	−0.1054	−0.0423	−0.1733
**Age‐standardized DALY rate**	473.8254	510.5076	437.2981	−0.0973	−0.0297	−0.1712
**Age‐standardized YLL rate**	442.1381	477.5181	409.0282	−0.1100	−0.0394	−0.1860
**Age‐standardized YLD rate**	31.6874	42.8056	22.1720	0.1288	0.2241	0.0443

*Note*: (a) ASIR, age‐standardized incidence rate. (b) ASDR, age‐standardized death rate. (c) DALY, disability adjusted life year. (d) YLL, years of life lost due to premature mortality. (e) YLD, years lived with disability.

**FIGURE 1:**
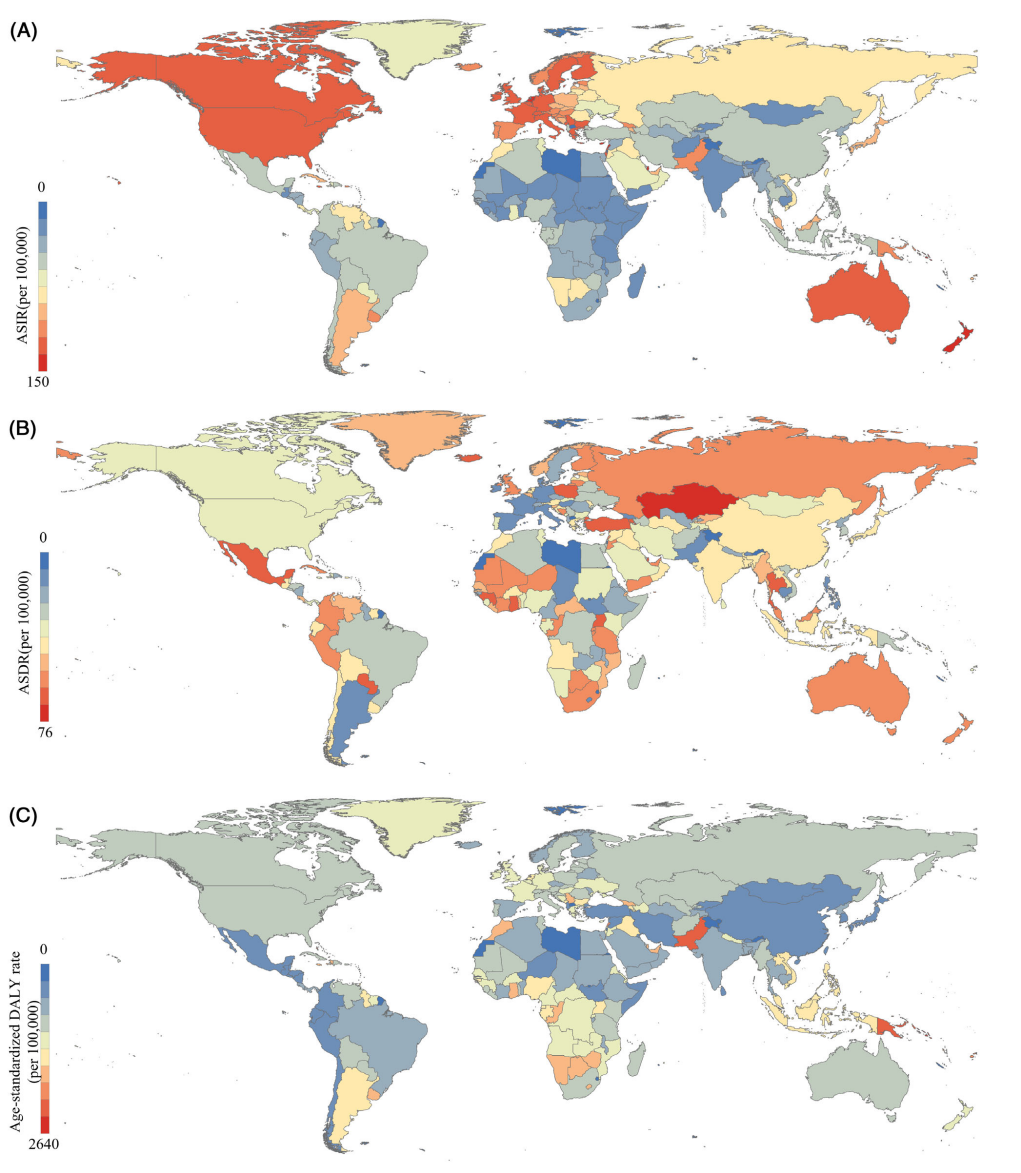
ASID, ASDR, and age‐standardized DALY rate for FBC by 204 countries, 2019. (Source: Figure 1 includes three figures, and the detailed subgroups are listed as follows: (A) ASIR; (B) ASDR; and (C) age‐standardized DALY rate)

Figure 1 shows some disease burden indicators for FBC in 2019 for the 204 countries. In terms of ASIR, the incidence was high in North America, Europe, and Oceania., whereas the incidence was relatively low in Asia, South America, and Africa. The ASIR varied widely by country, with Monaco (149.5994 per 100 000), the Solomon Islands (126.4800 per 100 000), Lebanon (122.5146 per 100 000), the Netherlands (111.4930 per 100 000), Qatar (103.7201 per 100 000), Barbados (102.3095 per 100 000), Cyprus (101.3340 per 100 000), and New Zealand (101.2075 per 100 000) all ranking higher in terms of incidence. As for ASDR, Asia, Oceania, and Africa had relatively high rates, while North America, South America, and Europe had relatively low rates. The death rate in Kazakhstan (75.0415/100000) was significantly higher than that in other countries. Meanwhile, Turkey (51.9397 per 100 000), Seychelles (43.4676 per 100 000), Ghana (42.1329 per 100 000), and Qatar (41.3708 per 100 000) ranked high in death rates. For age‐standardized DALY rates, the differences were comparatively small across regions, with relatively high DALYs in Africa and South America. The Solomon Islands (2635.7429 per 100 000) had a dramatically higher DALY rate than other countries. Moreover, Pakistan (1570.0613 per 100 000), Papua New Guinea (1467.9793 per 100 000), Micronesia (Federated States of Micronesia) (1238.2171 per 100 000), Nauru (1235.0406 per 100 000), and the Marshall Islands (1202.9005 per 100 000) ranked high for DALY rates. The YLL, YLD, and total percentage change for FBC are shown in supplemental file 3.

### 3.2 Age differences and risk factor differences in female breast cancer worldwide in 2019

**FIGURE 2:**
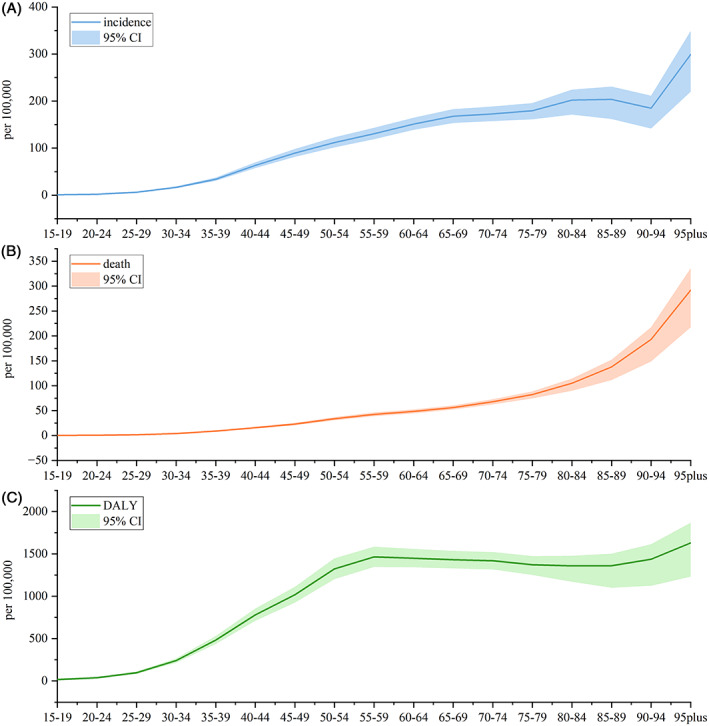
Incidence, death, and DALY rate for FBC by age, globally, 2019. (Source: Figure 2 includes three figures, and the detailed subgroups are listed as follows: (A) incidence, (B) death, and (C) DALY)

Figure 2 shows the global age‐specific disease burden indicators for FBC in 2019, starting from adolescents and continuing through the age of 95+ years. The incidence of FBC showed a trend of increasing with age, remaining stable between 80–90 years, and increasing faster after 90 years. The deaths due to FBC presented a growing trend with age, with the growth rate rising with age. The DALY rate of FBC appeared to go up with age, with a faster increase between 15 and 55 years of age, a steady state between 55 and 85 years, and a minor upside trend after 90 years.

**FIGURE 3:**
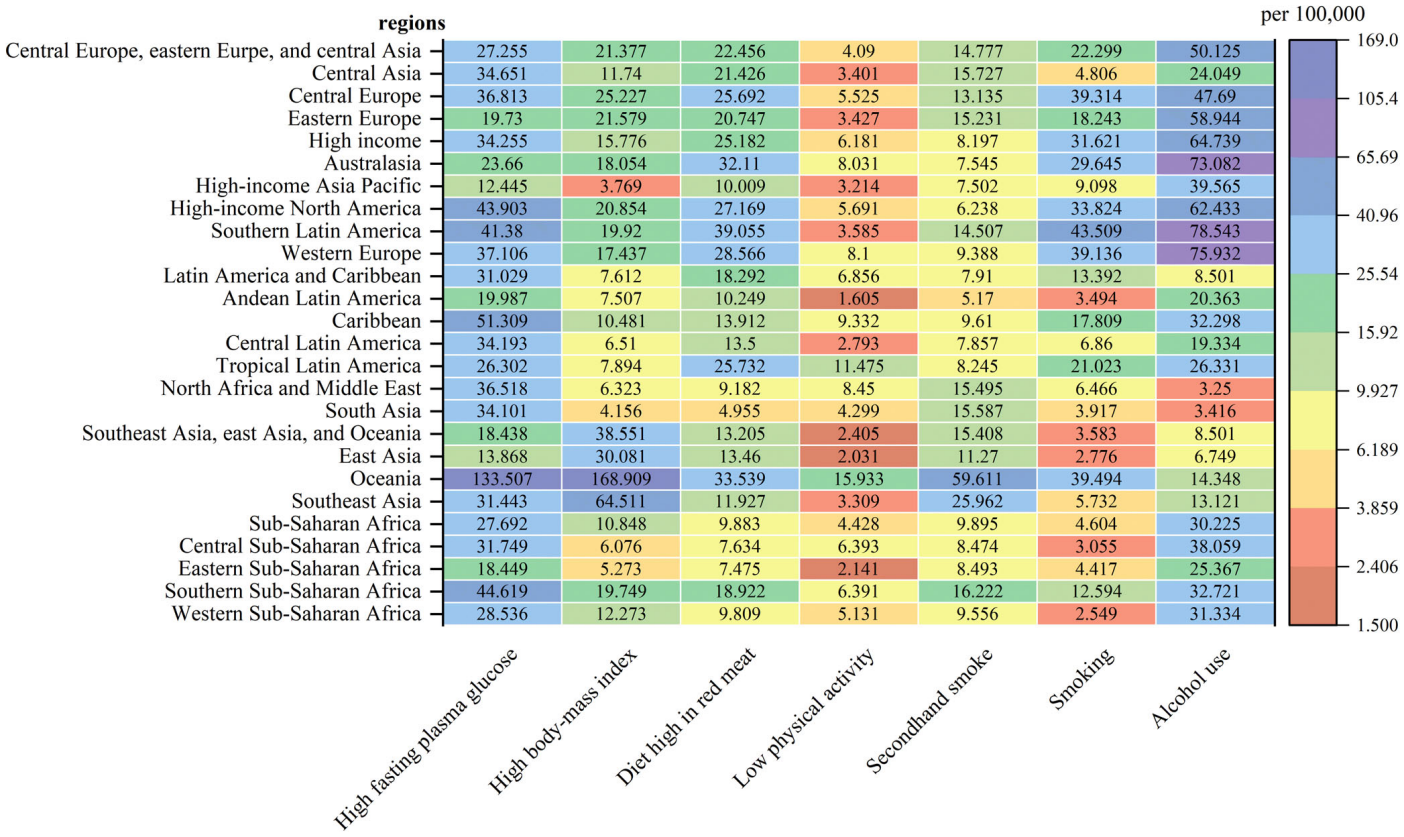
Age‐standardized DALY rate attributed to various risk factors in 21 GBD regions, 2019.

**FIGURE 4:**
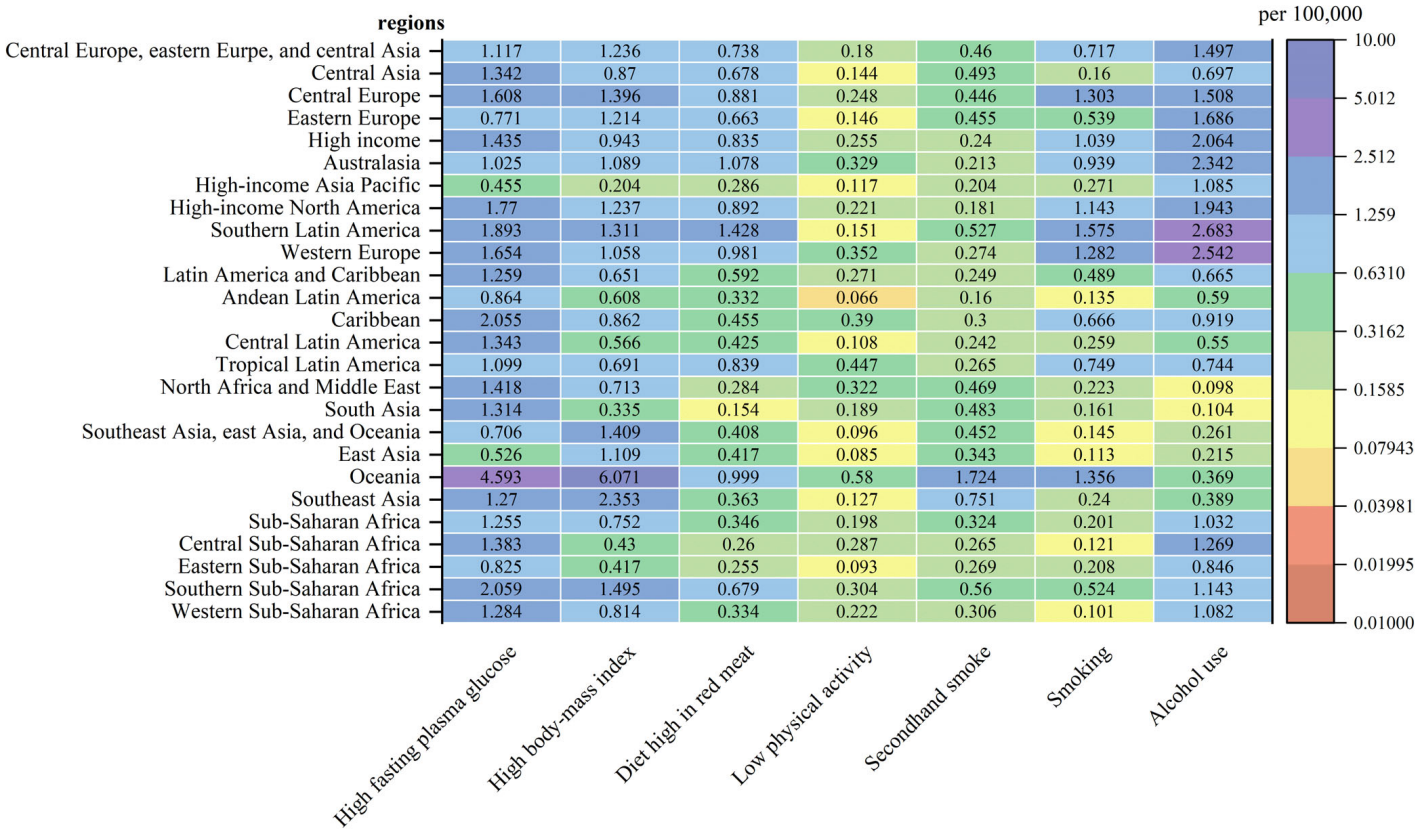
ASDR attributed to various risk factors in 21 GBD regions, 2019.

Figure 3 illustrates the DALY rates for various risk factors leading to FBC in each GBD region. Overall, alcohol use, high fasting plasma glucose, and high body mass index were the main risk factors for the DALYs of FBC. In some high‐income regions such as Europe, DALYs of FBC were mainly attributed to drinking. In Africa and the Americas, DALYs of FBC were mainly attributed to high fasting plasma glucose. In Oceania and Southeast Asia, DALYs of FBC were mainly attributed to high body mass index. Figure 4 shows the ASDR for the different risk factors leading to FBC in each GBD region, presenting similar characteristics to attributable DALYs.

### 3.3 Relationship between the incidence of female breast cancer and SDI

**FIGURE 5:**
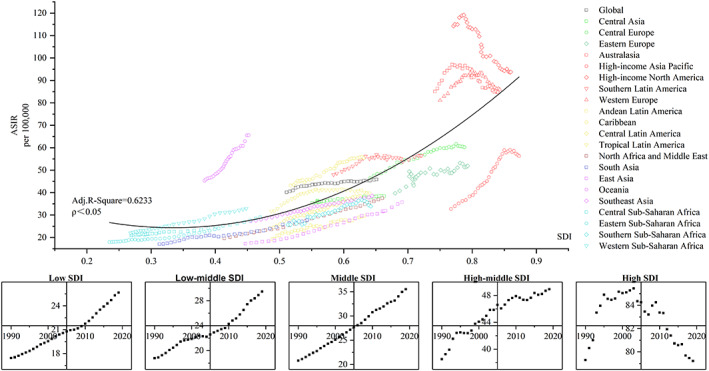
Age‐standardized DALY rates for FBC by 21 global burden of disease regions by Socio‐demographic Index (SDI), 1990–2019. (Source: (1) Figure 5 includes six figures, and the detailed subgroups are listed as follows: up: The relationship between ASIR and SDI globally and in the 21 GBD regions; down: The five figures represent the temporal trends of ASIR from 1990–2019 for Low SDI, Low‐middle SDI, Middle SDI, High‐middle SDI, and High SDI, respectively. (2) SDI, sociodemographic index; ASIR, age‐standardized incidence rate; Adj. R‐square, adjusted R‐square. (3) The solid black line represents expected values based on SDI from a regression of all location data over the entire 1990–2019 estimation period)

Figure 5 illustrates the relationship between the ASIR and SDI of the FBC. We selected an order of 2 for the polynomial fitting process (ρ < 0.05), and the adjusted R‐squared value was 0.6322, which was a good fit. The ASIR of the FBC followed an ascending trend with increasing SDI. The ASIR of Oceania, high‐income North America, Australasia, and Western Europe were significantly stronger than expected values, whereas that of high‐income Asia Pacific was significantly less. It is also interesting to note that within some high‐income regions, there is a tendency for ASIR to rise and then fall as SDI rises. Regarding the different levels of SDI regions, ASIR in the High SDI region presented a trend of initially rising and then falling with an increase in year, whereas the ASIR of other SDI regions continued to rise with increasing years.

### 3.4 Age‐specific and country‐specific projections of female breast cancer incidence in 2020–2044

**FIGURE 6:**
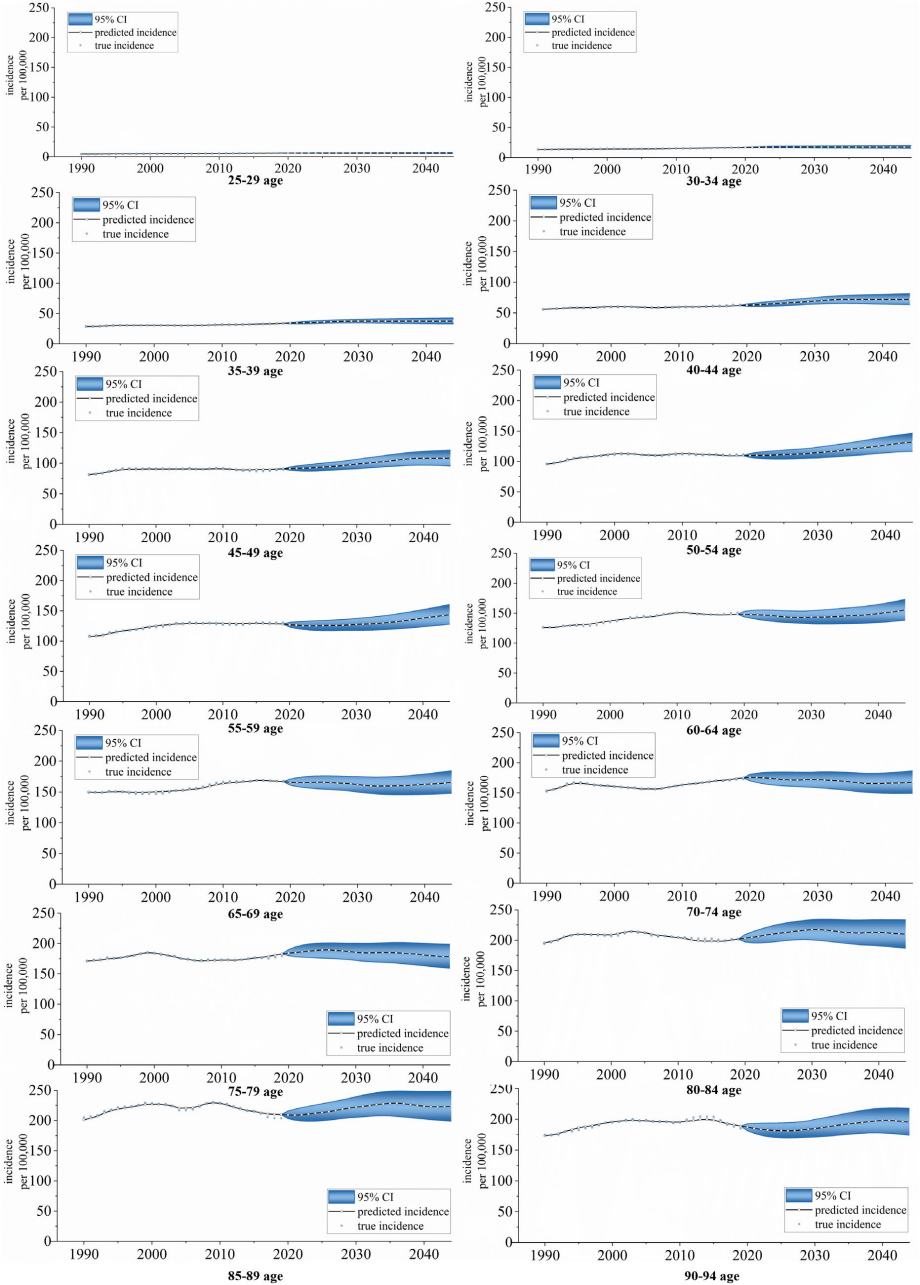
Incidence for FBC projections at the global level for 25–94‐year‐olds at five‐year age intervals, 2020–2044. (Source: Figure 1 includes 14 figures, and the detailed subgroups are listed as follows: from top to bottom, from left to right: 25–29 age group, 30–34 age group, 35–39 age group, 40–44 age group, 45–49 age group, 50–54 age group, 55–59 age group, 60–64 age group, 65–69 age group, 70–74 age group, 75–79 age group, 80–84 age group, 85–89 age group, and 90–94 age group)

Figure 6 shows age‐specific incidence projections for FBC at the global level for 25–94‐year‐olds in 2020–2044. The incidence of FBC is expected to vary worldwide by 2044 in all age groups. The incidences are 6.2562 (25–29 years, 0.18%), 17.6954 (30–34 years, 5.05%), 37.3583 (35–39 years, 10.41%), 71.9419 (40–44 years, 16.06%), 108.1527 (45–49 years, 19.00%), 131.5750 (50–54 years, 20.24%), 143.5679 (55–59 years, 11.91%), 155.5248 (60–64 years, 4.87%), 165.6904 (65–69 years, −0.62%), 167.7309 (70–74 years, −4.04%), 178.6400 (75–79 years, −2.24%), 210.0799 (80–84 years, 4.05%), 223.4441 (85–89 years, 6.54%), and 195.2365 (90–94 years, 3.35%) per 100 000, for the individual age groups. Supplemental file 4 contains the specific forecast data.

**FIGURE 7:**
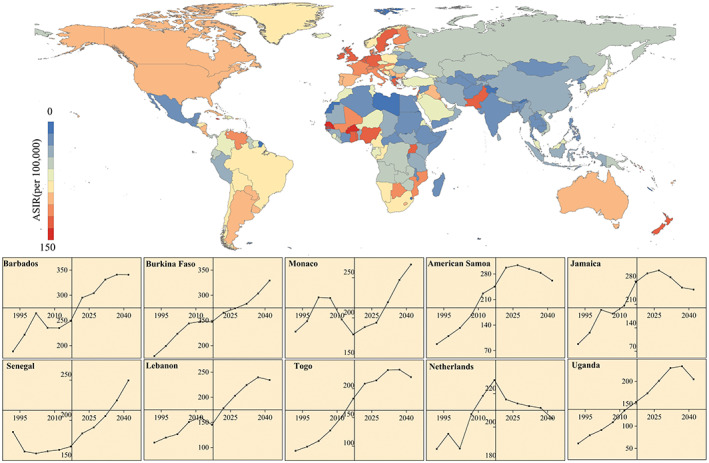
ASIR for FBC projections for 204 countries in 2040–2044 and temporal trends in countries with higher incidence rates in 1990–2044. (Source: Figure 7 includes 11 figures, and the detailed subgroups are listed as follows: up: The ASIR projections of FBC in 204 countries worldwide from 2040 to 2044; down: The ASIR projections of FBC for the top 10 countries in 2040–2044)

Figure 7 shows the ASIR projections of FBC for 204 countries worldwide for the years 2040–2044. The ASIR is expected to be relatively high in Europe, North America, and Oceania, followed by South America and Asia, and comparatively low in Africa. The degree of projected change in ASIR projections also varies across 204 countries. The top ASIR countries in 2044 are expected to be the COCOS (Keeling), (Islands) (353.4001 per 100 000), Barbados (340.9038 per 100 000), Burkina Faso (329.2729 per 100 000), Monaco (266.3889 per 100 000), American Samoa (261.8847 per 100 000), Jamaica (254.3641 per 100 000), Senegal (249.6059 per 100 000), Lebanon (234.4269 per 100 000), Togo (220.3110 per 100 000), the Netherlands (205.1530 per 100 000), and Uganda (204.9681 per 100 000). Most of these countries' ASIR is expected to show an upward trend in 2020–2044. However, American Samoa, Jamaica, and the Netherlands show a downward trend in ASIR. Supplemental file 5 shows the forecast results for the specific 204 countries.

**FIGURE 8:**
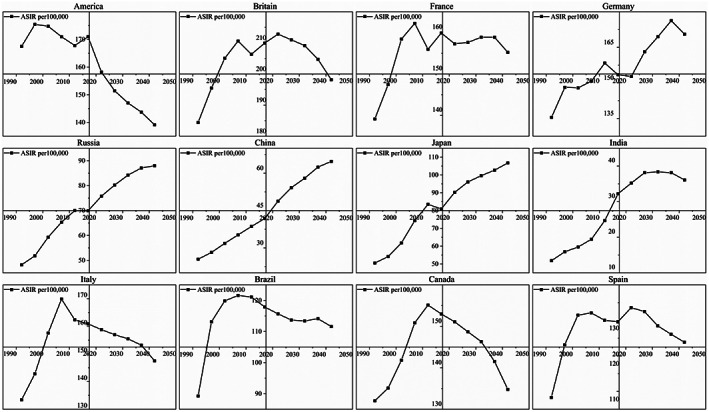
Temporal trends of ASIR in selected countries in 1990–2044. (Source: Figure 8 includes 12 figures, and the detailed subgroups are listed as follows: The ASIR of FBC for some selected countries from 1990 to 2044, from top to bottom, from left to right: America, Britain, France, Germany, Russia, China, Japan, India, Italy, Brazil, Canada, and Spain)

Figure 8 presents the ASIR trend for FBC in selected countries from 1990 to 2019 and the projected trend from 2020 to 2044. The ASIR for FBC in the US is expected to present a notable falling trend from 1990 to 2044. The UK, France, Italy, Brazil, Canada, and Spain will follow an upward and then downward trend. On the contrary, Germany, Russia, China, Japan, and India will consistently show a clear rising trend.

## 4 DISCUSSION

Although the worldwide incidence of FBC demonstrated a rising trend, the death rate showed a falling trend from 1990 to 2019, which is consistent with the findings of previous studies.^31^ This indicates that the survival rate of FBC is improving. Nevertheless, it is crucial to note that FBC patients often experience lymphedema,^32^ dyskinesia,^33^ induced amenorrhea,^34^ venous thromboembolism,^35^ persistent fatigue,^36^ and chronic pain.^37^ In the meantime, some patients may undergo mastectomy, which may lead to concerns about body image and sexuality.^38^ Therefore, psychological disorders are also a common problem for patients with FBC after surgery.^39^ In this context, we must focus on the rehabilitation of FBC survivors,^40^ prioritizing research development and testing of new interventions to reduce the symptoms and side effects of the disease.

The latest GBD 2019 data indicate that North America, Oceania, and Europe are areas with a high incidence of FBC. However, Asia and Africa have relatively high death rates. As a common cancer in women, the timely treatment of FBC can greatly affect the health outcomes of patients.^41^ Some studies^42^ have shown that socioeconomic status and treatment delay are related issues. Asia and Africa are relatively less economically developed than North America, Oceania, and Europe, and their patients may have longer delays in treatment, contributing to some of the differences in death rates across regions.

As for age structure, the worldwide disease burden of FBC increases with age, which is in accordance with the available studies.^43^ Meanwhile, we predicted the incidence of FBC showed a noticeable rising trend between 35 and 60 years, with the fastest rising rate in 50–54 years. Most randomized clinical trials and FBC screening guidelines recommend a uniform protocol, suggesting that all women should begin screening at 50 years old.^44^ However, when considered in conjunction with the predicted results of the risk of incidence by age group, it is necessary to adopt a risk‐appropriate age of screening onset and optimal time interval^45^ to ensure equity and validity of breast cancer screening claims.^46^ This finding suggests that focus should be placed on the detection and control of FBC in middle‐aged and older women.

In terms of risk factors, alcohol use^47^ and high fasting plasma glucose^48^ are the remarkable influential risk factors for FBC, which is in agreement with previous studies.^49^ Especially in Europe, the disease burden from alcohol use is even worse. As European countries have a traditional wine‐oriented diet,^50^ their total per capita alcohol consumption is higher than that of most developing countries,^51^ with the existence of alcohol dependence and abuse.^52^ The results of most epidemiological as well as experimental animal studies,^47^ suggest that alcohol intake can lead to the development of FBC through different mechanisms. In some African regions, the disease burden from high fasting plasma glucose is even worse. High fasting plasma glucose will likely develop into diabetes, which increases the risk of breast cancer. Diabetes is a major challenge faced by many African health systems.^53^ Studies^54^ have shown that over half of the cases of diabetes in Africa are undiagnosed. The results of the projection study^55^ indicated a steady upward trend in the prevalence of diabetes in Africa, increasing from 14.2 million in 2015 to 41 million in 2045. There are several complex causes possibly involved in the association of diabetes with breast cancer, including hyperglycemia, insulin signaling, insulin‐like growth factor 1 (IGF‐1) signaling, and regulation of endogenous sex hormones, which further increases the risk of breast cancer.^48^, ^56^, ^57^

From the relationship between ASIR and SDI, the results of our data corroborate that the ASIR of FBC increases with SDI, which is consistent with previous studies.^58^ The SDI^59^ is a comprehensive index of a country/region's development status and is influenced by indicators such as the overall fertility rate, the average education level of women, and per capita revenue. First, fertility affects ASIR in FBC because birth at any age briefly adds to the risk in the first decade postpartum.^60^ Second, economic disparities can affect the ASIR of FBC because women with low economic status are at risk of being diagnosed with FBC at a later stage.^61^, ^62^ This can lead to increased incidence over the long term. While national healthcare coverage is also greater in regions with better socioeconomic development, healthcare coverage can increase the screening rate for FBC patients.^63^, ^64^ We suggest that regions with significantly higher ASIR than the expected values should receive more resources for prevention and treatment, as represented by Oceania, while focusing on regions where ASIR grows faster with SDI than the expected speed, as represented by the Caribbean, Western Sub‐Saharan Africa, Central Sub‐Saharan Africa, and Eastern Sub‐Saharan Africa. This mainly focuses on the middle and low‐middle SDI regions. Especially in Africa, the prevalence of breast cancer screening is substantially low and varies gradually across countries.^65^ It is essential to vigorously promote a healthy lifestyle and strengthen female breast cancer screening^66^ to achieve a higher level of prevention in these regions.

We projected ASIR for 204 national FBCs from 2020 to 2044. In general, North America, Oceania, and Europe will remain high‐incidence areas for FBC in 2040–2044, whereas some countries in South America and Africa will face a considerable increase in incidence. The countries with high incidence that is expected to increase significantly are Barbados, Burkina Faso, Senegal, Monaco, Lebanon, Togo, and Uganda. Adherence to cancer prevention recommendations, such as screening, can reduce the risk of FBC.^67^ The association of fatalism with screening behavior may lead to a higher incidence in some racial/ethnic minority populations.^68^, ^69^, ^70^ The fatalism believed by some African‐American populations affects their cancer screening behavior and may reduce the use of screening mammograms by women,^71^ which makes it difficult to achieve FBC prevention. Regarding individual country forecasts, America, Britain, France, Italy, Brazil, Canada, and Spain have notably downward trends for 2020–2044. However, Germany, Russia, China, Japan, and Italy continue to show varying degrees of upward trends.

There are some limitations to our study. First, the GBD2019 reports estimated data. Owing to the poor data availability in some regions or countries, there may be bias between the reported and actual values. Therefore, there may be some statistical bias in our projections of future incidence. Second, owing to the lack of data, the current analysis could not explore the nature of the decline in ASIR in some high‐SDI regions. It is recommended that future studies focus on exploring FBC control strategies in high‐income North America, Australasia, and Western Europe to provide lessons for other regions. Third, Bayesian APC is based on historical data to predict incidence, which does not consider future changes in medical technology advances, national health policies, and other social factors. Although the results may have prediction bias, such long‐term predictions can be used to guide the management of future healthcare resource distribution and policy development.

The main finding is that FBC burden still varies between regions worldwide and that attention should be paid to its prevention and rehabilitation. The incidence among those aged 50–54 years is predicted to rise by approximately 20% in 2044, which warrants more focus on this age group. The countries with higher incidence and expected to increase significantly are Barbados, Burkina Faso, Senegal, Monaco, Lebanon, Togo, and Uganda. As the SDI is closely related to the ASIR of FBC, we suggest attaching importance to the control of middle and low‐middle SDI regions. In conclusion, public health and cancer prevention experts should pay more attention to regions and populations at increased risk of FBC while conducting further epidemiological studies to investigate the risk factors for increase in incidence.

## AUTHOR CONTRIBUTIONS

**Jingya Zhang:** Conceptualization (equal); data curation (equal); formal analysis (equal); investigation (equal); methodology (equal); resources (equal); software (equal); visualization (equal); writing – original draft (equal); writing – review and editing (equal). **Yongbo Lu:** Methodology (equal); software (equal); supervision (equal); validation (equal). **Ning Zhang:** Data curation (equal); supervision (equal); writing – review and editing (equal). **Zeru Yu:** Supervision (equal); validation (equal); writing – review and editing (equal). **Haoran Li:** Supervision (equal); validation (equal). **Rongxin He:** Supervision (equal); validation (equal). **Ying Mao:** Funding acquisition (equal); project administration (equal); supervision (equal). **Bin Zhu:** Conceptualization (equal); resources (equal); Supervision (equal); validation (equal); writing – review and editing (equal); Funding acquisition (equal); project administration (equal).

## FUNDING INFORMATION

This research was funded by Guangdong Provincial Natural Science Funds (grant number 2022A1515011871).

## CONFLICT OF INTEREST STATEMENT

The authors declare no potential competing interest.

## ETHICS STATEMENT

Not applicable. All the data we used are publicly available.

## SUPPLEMENTARY INFORMATION

Supplementary information accompanies this paper at https://doi.org/10.1186/XXXXXX.

## Supporting information


**Supplementary file 1:** Bayesian age‐period‐cohort model
**Supplementary file 2: Table S1.** ASIR, ASDR, and age‐standardized DALY rate for FBC in 2019 and percentage change of age‐standardized rates by location. **TableS2.** Age‐standardized YLL rate and age‐standardized YLD rate for FBC in 2019 and percentage change of age‐standardized rates by location.
**Supplementary file 3: Figure S1.** Age‐standardized YLL rate and age‐standardized YLD rate for FBC by 204 countries, 2019.
**Supplementary file 4: Table S3.** Incidence for FBC projections at the global level for 25–94 years at five‐year age intervals, 2020–2044 (25–59 age group). **Table S4.** Incidence for FBC projections at the global level for 25–94 years at five‐year age intervals, 2020–2044 (60‐94age group)
**Supplementary file 5: Figure S2‐206** The ASIR of FBC for 204 countries worldwide from 2019–2044.

## Data Availability

Data sharing is not applicable to this article as no new data were created or analyzed in this study.
