# Supplementary material for: Global burden of female breast cancer and its association with socioeconomic development status, 1990–2044
Source: Cancer Rep (Hoboken). 2023 Apr 24;6(Suppl 1):e1827. doi: 10.1002/cnr2.1827 (PMC10440843; doi:10.1002/cnr2.1827)
Supplement: Supplementary file 1 — Bayesian age‐period‐cohort model Supplementary file 2: Table S1. ASIR, ASDR, and age‐standardized DALY rate for FBC in 2019 and percentage change of age‐standardized rates by location. TableS2. Age‐standardized YLL rate and age‐standardized YLD rate for FBC in 2019 and percentage change of age‐standardized rates by location. Supplementary file 3: Figure S1. Age‐standardized YLL rate and age‐standardized YLD rate for FBC by 204 countries, 2019. Supplementary file 4: Table S3. Incidence for FBC projections at the global level for 25–94 years at five‐year age intervals, 2020–2044 (25–59 age group). Table S4. Incidence for FBC projections at the global level for 25–94 years at five‐year age intervals, 2020–2044 (60‐94age group) Supplementary file 5: Figure S2‐206 The ASIR of FBC for 204 countries worldwide from 2019–2044. [file CNR2-6-e1827-s001.docx]

# supplemental file1

## Bayesian age-period-cohort model

APC model is based on Poisson distribution, which improves the traditional descriptive analysis method. It decomposes the target analysis variables from three dimensions of age, period and cohort, allowing better report the risk of disease onset. The basic expression is:$\ln incidence=\mu+\alpha_{a}+\beta_{b}+\gamma_{c}+\varepsilon_{abc}$ where $\ln incidence$ represents the natural logarithm of the incidence, $\mu$ is the intercept term, $\alpha_{a}$ is the age effect in the a age group, $\beta_{b}$ is the period effect in the b age group, $\gamma_{c}$ is the cohort effect of the c birth cohort, $\varepsilon_{abc}$ is the error term or residual term. However, the linear relationship between these three components makes the complete model unidentifiable.

Bayesian APC assume that close time effects that are similar, attributing priors’ probability distributions. It is a hierarchical model that incorporates uncertainty about hyper parameters and avoids difficulties arising from the identifiability problem by the application of mildly informative prior distributions. It provides a method to calculate the probability of hypothesis, that is, according to the Bayesian formula, the prior information about unknown parameters and sample information are integrated to obtain posterior information, and then unknown parameters are inferred according to the posterior information. Among them, prior information comes from previous statistical conclusions, experiences or assumptions. Its formula is expressed as follows:

$$P\left( A_{i} \right)|B=\frac{P\left( A_{i} \right)P\left( B | A_{i} \right)}{\sum_{i}^{n} P\left( A_{i} \right)P\left( B | A_{i} \right)}(i=1,2,\ldots n)$$

Where $A_{1}$, $A_{2}$,..... $A_{n}$ are the complete event group of incompatible sample space, $P\left( A_{i} \right)$>0, P(B)>0.

The above formula is called Bayes' formula, where $P\left( A_{i} \right)$ represents the probability of the occurrence of $A_{i}$, namely the prior probability. This probability is a known probability, so the conditional probability of B occurring under the condition of $A_{i}$ occurring can be calculated according to the sample information, that is $P\left( B | A_{i} \right)$. Then, the probability $P\left( A_{i} \right)|B$ of the occurrence of condition A can be calculated according to the Bayesian formula under the condition of the occurrence of the resulting event B. This probability is the probability determined after the test, that is the posterior probability.

It is very difficult to determine the posteriori probability distribution type when applying Bayes formula, especially when the posteriori probability distribution is complicated. With the development of computer, the method based on MCMC simulation can solve the problem that the posterior probability is difficult to determine. The MCMC method is to obtain the approximate solution of the problem by simulating random events repeatedly and statistically analyzing the simulation results. Therefore, the error can be reduced by increasing the simulation times. When solving APC model parameters, the method based on MCMC simulation can be considered. When solving APC model parameters by MCMC simulation, the distribution of each parameter can be set according to the situation of sample data. By smoothing the effects of age, period and cohort, large fluctuations between two adjacent groups can be avoided in the estimation results, making the estimation results more robust and reliable.

BAMP software package can be used for modeling and prediction. BAMP software is written by Volker Sehmid and Leonhard Held based on a Bayesian age-period method. Queue models are used to analyze and predict illness or death in software. The software can also be applied to data types with different ages and periods. The principle of software simulation is to use Markov chain Monte Carlo method to iterate, so that the posterior probability distribution converges to a relatively stable state, and then to estimate the parameter values through the estimated samples obtained by iteration. Therefore, the more iterations, the higher the accuracy of model fitting. Generally, the number of iterations set in the study is 1.01 million, and the first 10,000 results of the initial iteration are omitted to eliminate the influence of artificially set initial values on the results. After that, one result is selected for every 500 iterations to form the sample of parameter estimation.

# supplemental file2

## ASIR, ASDR, and age-standardized DALY rate for FBC in 2019 and percentage change of age-standardized rates by location.

| **Region** | **ASIR**  **(Per 100,000)** | | | **Percentage change in age-standardised rates, 1990–2019** | | | **ASDR**  **(Per 100,000)** | | | **Percentage change in age-standardised rates, 1990–2019** | | | **age-standardized** | | | **Percentage change in age-standardised rates, 1990–2019** | | |
| --- | --- | --- | --- | --- | --- | --- | --- | --- | --- | --- | --- | --- | --- | --- | --- | --- | --- | --- |
|  |  |  |  |  |  |  |  |  |  |  |  |  | **DALY rate** | | |  |  |  |
|  |  |  |  |  |  |  |  |  |  |  |  |  | **(Per 100,000)** | | |  |  |  |
|  | **2019 value** | **Upper** | **Lower** | **Value** | **Upper** | **Lower** | **2019 value** | **Upper** | **Lower** | **Value** | **Upper** | **Lower** | **2019 value** | **Upper** | **Lower** | **Value** | **Upper** | **Lower** |
| Global | 45.8568 | 49.7581 | 41.9079 | 0.1431 | 0.2398 | 0.0475 | 15.8838 | 17.0711 | 14.6557 | -0.1054 | -0.0423 | -0.1733 | 473.8254 | 510.5076 | 437.2981 | -0.0973 | -0.0297 | -0.1712 |
| High SDI | 79.2239 | 87.6977 | 70.8267 | -0.001 | 0.1068 | -0.105 | 16.7083 | 17.4525 | 15.5603 | -0.3 | -0.2748 | -0.3264 | 487.4549 | 518.8382 | 459.7601 | -0.3067 | -0.2788 | -0.3342 |
| High-middle SDI | 48.9344 | 54.4861 | 43.8353 | 0.2703 | 0.422 | 0.1376 | 14.9347 | 16.1875 | 13.755 | -0.1543 | -0.0802 | -0.2148 | 434.9609 | 473.3057 | 400.6948 | -0.1859 | -0.112 | -0.2493 |
| Middle-SDI | 35.5168 | 39.8075 | 31.4716 | 0.7071 | 0.9556 | 0.4858 | 13.6593 | 15.183 | 12.303 | 0.0778 | 0.2121 | -0.0515 | 422.9095 | 468.867 | 378.6206 | 0.0585 | 0.1886 | -0.0709 |
| Low-middle SDI | 29.4725 | 33.1971 | 25.9068 | 0.5676 | 0.8839 | 0.3066 | 16.859 | 19.2388 | 14.5933 | 0.2071 | 0.481 | -0.0222 | 523.521 | 597.2068 | 452.0876 | 0.1995 | 0.4517 | -0.0173 |
| Low SDI | 25.6694 | 29.0963 | 22.5445 | 0.4723 | 0.8248 | 0.2003 | 18.3423 | 20.8386 | 15.985 | 0.2717 | 0.5779 | 0.0197 | 544.0272 | 621.5632 | 475.6096 | 0.2422 | 0.5169 | -0.0006 |
| **Central Europe, eastern Eurpe, and central Asia** | 52.4322 | 58.9769 | 47.3002 | 0.2738 | 0.4234 | 0.1523 | 18.3468 | 20.3766 | 16.6607 | -0.0405 | 0.0573 | -0.122 | 539.0718 | 599.9639 | 489.3271 | -0.107 | -0.014 | -0.1828 |
| Central Asia | 38.3637 | 42.8004 | 34.2307 | 0.0838 | 0.2205 | -0.0336 | 17.295 | 19.1608 | 15.5338 | -0.0838 | 0.0164 | -0.1744 | 523.8827 | 588.6651 | 464.1193 | -0.1448 | -0.0394 | -0.2364 |
| Central Europe | 60.2198 | 69.5654 | 52.0405 | 0.2972 | 0.4819 | 0.1334 | 19.8691 | 22.7064 | 17.2522 | -0.0876 | 0.0353 | -0.2029 | 552.6638 | 638.6561 | 476.9545 | -0.1457 | -0.0242 | -0.2588 |
| Eastern Europe | 51.8908 | 61.3102 | 44.1408 | 0.3075 | 0.5307 | 0.1149 | 17.4679 | 20.3553 | 15.0513 | -0.0257 | 0.1274 | -0.1526 | 529.1322 | 618.5698 | 456.07 | -0.0887 | 0.0623 | -0.2079 |
| **High income** | 80.899 | 89.9257 | 71.7864 | 0.0069 | 0.1223 | -0.1034 | 17.4076 | 18.1507 | 16.2163 | -0.2938 | -0.2704 | -0.3176 | 505.0972 | 536.495 | 477.357 | -0.3065 | -0.28 | -0.3309 |
| Australasia | 84.6912 | 104.9909 | 68.2527 | -0.0039 | 0.2366 | -0.1981 | 17.4676 | 18.6886 | 16.1077 | -0.3399 | -0.2964 | -0.3817 | 509.0317 | 552.7786 | 471.0526 | -0.3592 | -0.3111 | -0.4028 |
| High-income Asia Pacific | 56.2969 | 67.1838 | 47.1371 | 0.7193 | 1.0445 | 0.4432 | 9.7789 | 10.4066 | 8.9052 | 0.1231 | 0.182 | 0.058 | 321.9386 | 349.1726 | 300.0999 | 0.0909 | 0.1556 | 0.0325 |
| High-income North America | 93.7503 | 112.6412 | 78.0251 | -0.1792 | -0.0066 | -0.3151 | 18.3568 | 19.1935 | 17.275 | -0.3336 | -0.3089 | -0.3595 | 533.815 | 569.7994 | 502.4863 | -0.3634 | -0.3326 | -0.3923 |
| Southern Latin America | 56.5068 | 71.9353 | 43.7836 | 0.1775 | 0.487 | -0.0843 | 24.0411 | 25.6092 | 22.4115 | -0.1603 | -0.1082 | -0.2129 | 643.6675 | 691.6117 | 602.2981 | -0.1902 | -0.1332 | -0.2452 |
| Western Europe | 85.8526 | 98.8465 | 74.1247 | 0.0588 | 0.2208 | -0.0807 | 19.7905 | 20.7658 | 18.3211 | -0.3024 | -0.2757 | -0.3322 | 552.5588 | 591.6793 | 519.7774 | -0.3323 | -0.3003 | -0.3617 |
| **Latin America and Caribbean** | 39.587 | 44.0395 | 35.9122 | 0.3671 | 0.5272 | 0.2444 | 14.5047 | 15.9052 | 13.2431 | -0.0658 | 0.0244 | -0.1436 | 432.5964 | 477.0821 | 392.8487 | -0.0607 | 0.0382 | -0.1434 |
| Andean Latin America | 29.625 | 36.4463 | 24.0463 | 0.5417 | 0.9477 | 0.2096 | 12.6743 | 15.5053 | 10.4436 | -0.0047 | 0.2417 | -0.2046 | 370.3499 | 459.5088 | 300.1853 | -0.0387 | 0.2206 | -0.2364 |
| Caribbean | 55.3673 | 65.1083 | 46.6302 | 0.283 | 0.5043 | 0.0933 | 20.8374 | 24.3953 | 17.617 | 0.0407 | 0.2105 | -0.1089 | 623.6189 | 740.8605 | 515.1883 | 0.0449 | 0.2283 | -0.121 |
| Central Latin America | 38.4531 | 45.6353 | 32.2974 | 0.5981 | 0.906 | 0.3478 | 12.8746 | 15.0877 | 11.051 | 0.0398 | 0.2125 | -0.1084 | 390.0838 | 460.4123 | 330.638 | 0.0477 | 0.2303 | -0.1097 |
| Tropical Latin America | 39.7452 | 42.2402 | 37.2399 | 0.2526 | 0.3392 | 0.175 | 15.1886 | 16.1139 | 14.1487 | -0.1438 | -0.0873 | -0.1942 | 451.1776 | 479.0937 | 424.3605 | -0.1355 | -0.0799 | -0.1862 |
| **North Africa and Middle East** | 37.4834 | 42.9358 | 32.6773 | 0.9087 | 1.2214 | 0.5456 | 15.2226 | 17.3465 | 13.3092 | 0.2398 | 0.4558 | -0.0084 | 472.7338 | 544.7538 | 409.0018 | 0.1954 | 0.4046 | -0.0317 |
| **South Asia** | 27.7246 | 33.0002 | 22.9079 | 0.6258 | 1.1336 | 0.2374 | 16.8258 | 19.9956 | 13.9051 | 0.2551 | 0.6764 | -0.0598 | 520.5907 | 620.4408 | 426.8429 | 0.2818 | 0.6823 | -0.0298 |
| **Southeast Asia, east Asia, and Oceania** | 36.6669 | 43.2403 | 30.8267 | 0.8535 | 1.2795 | 0.4905 | 11.7779 | 13.5131 | 10.275 | 0.0162 | 0.2027 | -0.1466 | 376.9138 | 430.7983 | 329.1298 | -0.0053 | 0.1801 | -0.1673 |
| East Asia | 35.6859 | 44.5369 | 28.3163 | 1.0711 | 1.7376 | 0.535 | 9.1209 | 11.1263 | 7.3561 | -0.0086 | 0.2793 | -0.2357 | 282.1524 | 341.1918 | 230.8063 | -0.046 | 0.2461 | -0.259 |
| Oceania | 65.5768 | 83.5796 | 50.4366 | 0.4486 | 0.888 | 0.1074 | 42.7956 | 54.2313 | 33.194 | 0.3041 | 0.7004 | -0.0004 | ######## | ######## | ######## | 0.3039 | 0.7302 | -0.0156 |
| Southeast Asia | 38.5203 | 44.6419 | 33.1132 | 0.409 | 0.6934 | 0.1548 | 19.2299 | 22.005 | 16.6179 | 0.021 | 0.2086 | -0.156 | 621.2223 | 719.1038 | 534.0738 | -0.0036 | 0.1927 | -0.1889 |
| **Sub-Saharan Africa** | 29.5437 | 34.0479 | 25.265 | 0.4086 | 0.705 | 0.1249 | 21.2978 | 24.5613 | 18.1868 | 0.2511 | 0.521 | -0.0053 | 589.2356 | 691.5558 | 491.8611 | 0.1945 | 0.4566 | -0.0514 |
| Central Sub-Saharan Africa | 28.9777 | 38.5511 | 20.858 | 0.3837 | 0.8867 | -0.0037 | 22.4198 | 29.7634 | 16.1634 | 0.2565 | 0.6954 | -0.0871 | 627.5309 | 827.7681 | 452.4599 | 0.2146 | 0.6587 | -0.1288 |
| Eastern Sub-Saharan Africa | 24.0446 | 27.4866 | 20.7755 | 0.3399 | 0.6416 | 0.0621 | 18.1532 | 20.5971 | 15.6453 | 0.196 | 0.4549 | -0.0342 | 501.5443 | 580.6389 | 427.7228 | 0.1203 | 0.3942 | -0.099 |
| Southern Sub-Saharan Africa | 33.8912 | 38.0185 | 30.1417 | 0.3201 | 0.5168 | 0.138 | 22.0566 | 24.5363 | 19.7207 | 0.1946 | 0.3775 | 0.037 | 588.0307 | 667.0811 | 517.7414 | 0.1042 | 0.2757 | -0.0409 |
| Western Sub-Saharan Africa | 32.9138 | 40.1108 | 25.9252 | 0.4995 | 0.9865 | 0.0715 | 23.2468 | 28.6228 | 18.6507 | 0.3133 | 0.7156 | -0.0331 | 650.9567 | 818.7377 | 506.4112 | 0.2672 | 0.676 | -0.0719 |

## Age-standardized YLL rate and age-standardized YLD rate for FBC in 2019 and percentage change of age-standardized rates by location.

| **Region** | **Age-standardized YLL rate**  **(Per 100,000)** | | | **Percentage change in age-standardized rates, 1990–2019** | | | **Age-standardized YLD rate**  **(Per 100,000)** | | | **Percentage change in age-standardized rates, 1990–2019** | | |
| --- | --- | --- | --- | --- | --- | --- | --- | --- | --- | --- | --- | --- |
|  | **2019 value** | **Upper** | **Lower** | **value** | **Upper** | **Lower** | **2019 value** | **Upper** | **Lower** | **value** | **Upper** | **Lower** |
| Global | 442.1381 | 477.5181 | 409.0282 | -0.1100 | -0.0394 | -0.1860 | 31.6874 | 42.8056 | 22.1720 | 0.1288 | 0.2241 | 0.0443 |
| High SDI | 427.9008 | 443.8952 | 409.2940 | -0.3361 | -0.3141 | -0.3597 | 59.5541 | 80.6048 | 40.9030 | 0.0174 | 0.1201 | -0.0770 |
| High-middle SDI | 400.8142 | 436.9074 | 369.5645 | -0.2102 | -0.1375 | -0.2746 | 34.1467 | 46.5464 | 23.5395 | 0.2735 | 0.4202 | 0.1492 |
| Middle-SDI | 399.7884 | 444.8949 | 359.1167 | 0.0345 | 0.1642 | -0.0954 | 23.1211 | 31.6237 | 16.1991 | 0.7679 | 1.0303 | 0.5429 |
| Low-middle SDI | 505.9069 | 579.0538 | 436.7950 | 0.1887 | 0.4448 | -0.0307 | 17.6141 | 23.6639 | 12.6766 | 0.6220 | 0.9080 | 0.3802 |
| Low SDI | 529.5764 | 607.3582 | 461.7574 | 0.2364 | 0.5133 | -0.0050 | 14.4508 | 19.3447 | 10.2561 | 0.5027 | 0.8298 | 0.2366 |
| **Central Europe, Eastern Europe, and Central Asia** | 503.2339 | 562.3872 | 456.5650 | -0.1261 | -0.0312 | -0.2037 | 35.8379 | 49.1575 | 24.9883 | 0.2888 | 0.4235 | 0.1722 |
| Central Asia | 498.3782 | 558.6554 | 443.1133 | -0.1545 | -0.0537 | -0.2456 | 25.5045 | 35.1163 | 18.0623 | 0.1021 | 0.2322 | -0.0137 |
| Central Europe | 511.5939 | 590.3878 | 441.3082 | -0.1692 | -0.0486 | -0.2834 | 41.0699 | 56.2420 | 28.5292 | 0.3189 | 0.4973 | 0.1583 |
| Eastern Europe | 493.4215 | 579.1621 | 420.3661 | -0.1088 | 0.0454 | -0.2355 | 35.7107 | 50.8521 | 24.0942 | 0.3215 | 0.5246 | 0.1395 |
| **High-income** | 443.9233 | 459.4217 | 426.0602 | -0.3362 | -0.3155 | -0.3558 | 61.1739 | 83.2536 | 42.2566 | 0.0273 | 0.1362 | -0.0729 |
| Australasia | 444.1650 | 470.7507 | 415.7772 | -0.3917 | -0.3560 | -0.4281 | 64.8667 | 93.1986 | 43.7318 | 0.0094 | 0.2298 | -0.1755 |
| High-income Asia Pacific | 278.0966 | 293.0239 | 262.0695 | 0.0364 | 0.0867 | -0.0131 | 43.8421 | 62.6705 | 29.1886 | 0.6377 | 0.9309 | 0.4056 |
| High-income North America | 463.9740 | 481.7510 | 444.4912 | -0.3863 | -0.3626 | -0.4105 | 69.8410 | 97.7434 | 46.8999 | -0.1536 | 0.0065 | -0.2828 |
| Southern Latin America | 604.1456 | 642.2619 | 568.8349 | -0.2079 | -0.1548 | -0.2582 | 39.5219 | 56.4545 | 25.9838 | 0.2272 | 0.5356 | -0.0353 |
| Western Europe | 487.0963 | 507.1042 | 463.3597 | -0.3649 | -0.3410 | -0.3884 | 65.4625 | 91.4048 | 44.6999 | 0.0811 | 0.2249 | -0.0484 |
| **Latin America and Caribbean** | 408.9093 | 451.6060 | 371.9978 | -0.0790 | 0.0179 | -0.1613 | 23.6871 | 31.9475 | 16.9876 | 0.4278 | 0.5899 | 0.2958 |
| Andean Latin America | 352.9436 | 438.1123 | 283.6109 | -0.0580 | 0.2005 | -0.2524 | 17.4063 | 24.3587 | 11.8788 | 0.6414 | 1.0765 | 0.2939 |
| Caribbean | 590.4213 | 702.4060 | 489.0881 | 0.0337 | 0.2188 | -0.1313 | 33.1976 | 45.1684 | 23.1552 | 0.2942 | 0.5121 | 0.0976 |
| Central Latin America | 367.0248 | 431.7030 | 310.2306 | 0.0241 | 0.2037 | -0.1340 | 23.0590 | 31.8966 | 16.2572 | 0.6561 | 0.9513 | 0.4051 |
| Tropical Latin America | 427.3839 | 453.3704 | 402.8557 | -0.1521 | -0.0955 | -0.2030 | 23.7937 | 31.7583 | 17.1295 | 0.3307 | 0.4315 | 0.2363 |
| **North Africa and Middle East** | 448.1895 | 516.6430 | 386.2661 | 0.1710 | 0.3742 | -0.0528 | 24.5443 | 33.4575 | 17.2055 | 0.9301 | 1.2500 | 0.5857 |
| **South Asia** | 504.2382 | 604.4668 | 411.8359 | 0.2717 | 0.6793 | -0.0393 | 16.3524 | 22.6810 | 11.2258 | 0.6968 | 1.1645 | 0.3372 |
| **Southeast Asia, East Asia, and Oceania** | 352.0562 | 404.8161 | 305.0395 | -0.0380 | 0.1457 | -0.2038 | 24.8576 | 34.7576 | 16.5344 | 0.9224 | 1.3377 | 0.5679 |
| East Asia | 257.5090 | 317.5056 | 206.0158 | -0.0935 | 0.1943 | -0.3142 | 24.6434 | 34.9506 | 16.0696 | 1.1058 | 1.7257 | 0.6059 |
| Oceania | 1378.3202 | 1756.4559 | 1054.7488 | 0.3000 | 0.7288 | -0.0189 | 38.5542 | 54.1605 | 25.5787 | 0.4595 | 0.8772 | 0.1144 |
| Southeast Asia | 596.4416 | 692.1969 | 509.2703 | -0.0174 | 0.1810 | -0.2003 | 24.7807 | 34.7372 | 17.2785 | 0.5023 | 0.7829 | 0.2478 |
| **Sub-Saharan Africa** | 572.7055 | 672.7123 | 478.0319 | 0.1887 | 0.4518 | -0.0581 | 16.5301 | 22.3072 | 11.5869 | 0.4355 | 0.7089 | 0.1703 |
| Central Sub-Saharan Africa | 611.8772 | 806.9694 | 441.3001 | 0.2105 | 0.6507 | -0.1345 | 15.6538 | 22.6882 | 9.9539 | 0.4003 | 0.9186 | 0.0102 |
| Eastern Sub-Saharan Africa | 488.2243 | 564.6577 | 416.0721 | 0.1147 | 0.3874 | -0.1036 | 13.3200 | 17.8233 | 9.3436 | 0.3700 | 0.6810 | 0.0833 |
| Southern Sub-Saharan Africa | 568.4518 | 644.4412 | 500.0045 | 0.0981 | 0.2701 | -0.0482 | 19.5789 | 26.0588 | 13.8340 | 0.3152 | 0.5000 | 0.1508 |
| Western Sub-Saharan Africa | 632.4263 | 800.3414 | 492.5826 | 0.2606 | 0.6765 | -0.0796 | 18.5305 | 26.1002 | 12.4379 | 0.5427 | 1.0213 | 0.1273 |

# supplemental file3

## Age-standardized YLL rate and age-standardized YLD rate for FBC by 204 countries, 2019.


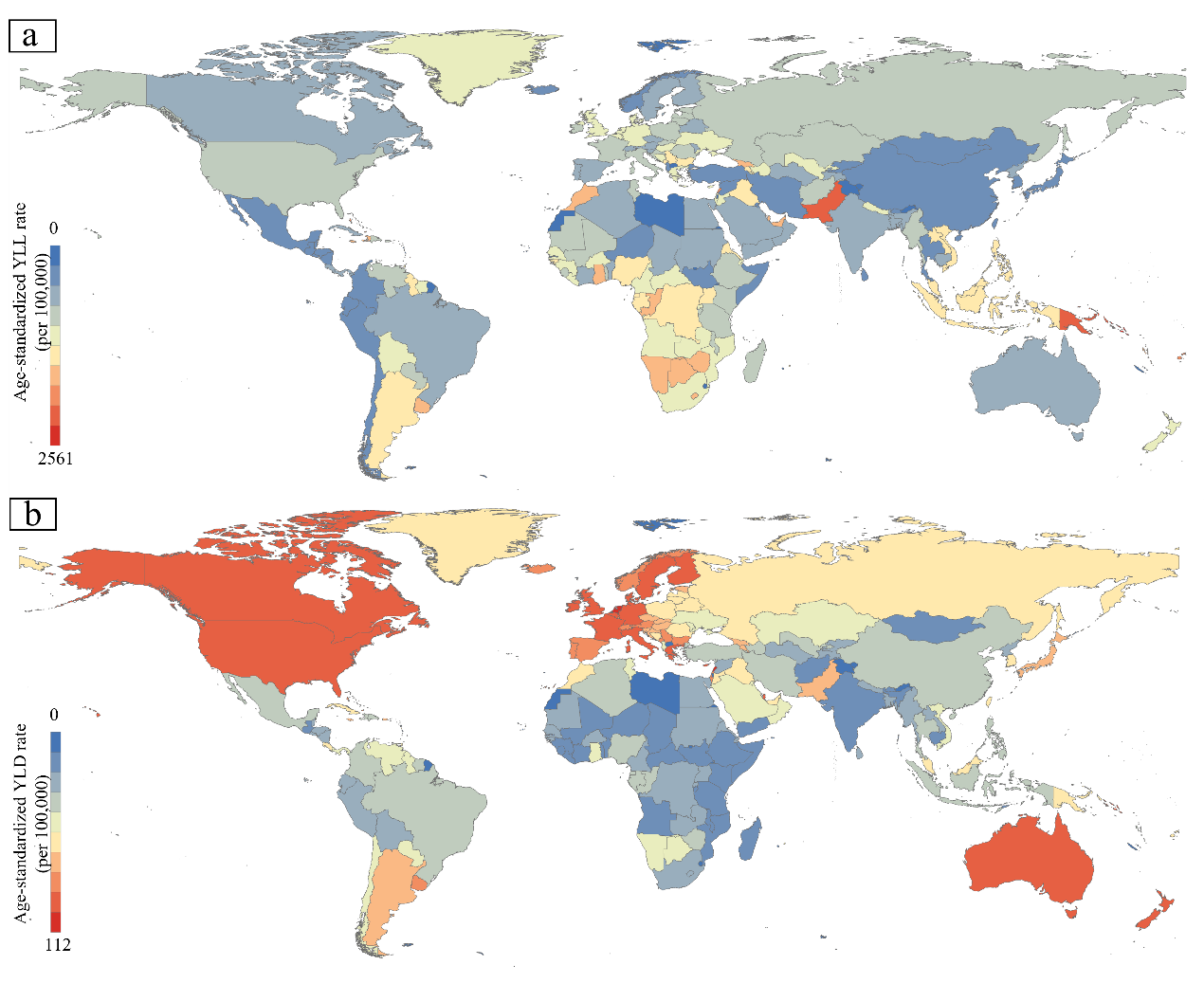


# supplemental file4

## Incidence for FBC projections at the global level for 25-94 years at five-year age intervals, 2020-2044 (25-59 age group)

| **year** | **25-29** | | **30-34** | | **35-39** | | **40-44** | | **45-49** | | **50-54** | | **55-59** | |
| --- | --- | --- | --- | --- | --- | --- | --- | --- | --- | --- | --- | --- | --- | --- |
|  | **GBD value** | **Predicted value** | **GBD value** | **Predicted value** | **GBD value** | **Predicted value** | **GBD value** | **Predicted value** | **GBD value** | **Predicted value** | **GBD value** | **Predicted value** | **GBD value** | **Predicted value** |
| **1990** | 4.3616 | 4.6482 | 13.3741 | 13.4622 | 28.6630 | 28.4471 | 55.7164 | 55.9145 | 80.9432 | 81.6119 | 95.9614 | 95.4581 | 108.7959 | 107.2992 |
| **1991** | 4.3544 | 4.6535 | 13.5373 | 13.5349 | 28.9522 | 28.6062 | 56.4013 | 56.4243 | 81.8689 | 82.8236 | 97.7771 | 97.0236 | 109.5703 | 108.2579 |
| **1992** | 4.3686 | 4.6796 | 13.5869 | 13.6426 | 29.2228 | 28.8891 | 56.7011 | 56.8661 | 83.4786 | 84.3411 | 99.7515 | 98.9388 | 110.7211 | 109.5763 |
| **1993** | 4.4163 | 4.7640 | 13.7413 | 13.8562 | 30.0112 | 29.5294 | 57.8260 | 57.6608 | 86.2649 | 86.5070 | 103.5957 | 101.8853 | 114.5091 | 112.5678 |
| **1994** | 4.6320 | 4.8352 | 14.0940 | 14.0145 | 30.4909 | 30.0442 | 58.0095 | 58.1636 | 89.2609 | 88.2005 | 105.5655 | 104.2472 | 115.9708 | 114.8809 |
| **1995** | 4.8423 | 4.8928 | 14.3574 | 14.0552 | 30.7762 | 30.3409 | 57.7087 | 58.3693 | 91.6526 | 89.6038 | 107.0461 | 106.1386 | 116.8092 | 116.8479 |
| **1996** | 4.9379 | 4.9323 | 14.3900 | 14.0213 | 30.6422 | 30.3965 | 57.5560 | 58.4668 | 91.9532 | 90.1433 | 107.5929 | 107.1569 | 117.7024 | 118.1992 |
| **1997** | 5.0224 | 4.9493 | 14.4753 | 14.0014 | 30.6559 | 30.4194 | 57.7889 | 58.6599 | 91.5279 | 90.0553 | 108.5615 | 108.3619 | 118.7847 | 119.6991 |
| **1998** | 5.1836 | 4.9972 | 14.6862 | 14.0598 | 30.5544 | 30.5217 | 58.4254 | 59.2017 | 91.4545 | 90.2588 | 108.8188 | 109.8309 | 119.8067 | 121.7686 |
| **1999** | 5.2750 | 5.0722 | 14.8007 | 14.1905 | 30.5114 | 30.6914 | 59.2643 | 59.8340 | 91.7425 | 90.5141 | 109.7999 | 111.2915 | 122.0004 | 123.8447 |
| **2000** | 5.3454 | 5.1215 | 14.9201 | 14.2745 | 30.5235 | 30.5456 | 59.4331 | 60.0228 | 91.2398 | 90.3042 | 111.1546 | 112.4025 | 123.2093 | 125.2927 |
| **2001** | 5.4453 | 5.1574 | 15.0562 | 14.3711 | 30.6248 | 30.4684 | 59.6177 | 60.1541 | 91.5689 | 90.3415 | 111.8710 | 112.8094 | 124.0125 | 126.4103 |
| **2002** | 5.2849 | 5.1790 | 14.4969 | 14.3841 | 30.3756 | 30.3784 | 59.8441 | 59.9925 | 90.8391 | 90.4629 | 111.9304 | 112.6060 | 127.3322 | 127.3680 |
| **2003** | 5.1505 | 5.2036 | 14.1558 | 14.4451 | 30.0624 | 30.2636 | 59.7082 | 59.7936 | 90.6612 | 90.5858 | 112.2184 | 112.1385 | 129.9490 | 128.3532 |
| **2004** | 5.1769 | 5.2213 | 14.1698 | 14.4589 | 29.9223 | 30.1709 | 59.0957 | 59.3761 | 91.2070 | 90.4486 | 110.7726 | 110.8422 | 129.5840 | 128.3660 |
| **2005** | 5.2614 | 5.2841 | 14.2155 | 14.5695 | 30.2361 | 30.2949 | 59.2784 | 59.0819 | 92.1807 | 90.6326 | 110.4858 | 110.4404 | 131.1331 | 129.3674 |
| **2006** | 5.2744 | 5.3199 | 14.1421 | 14.5981 | 30.2745 | 30.3413 | 58.8554 | 58.5262 | 90.8585 | 90.2972 | 109.3896 | 109.9545 | 130.0672 | 129.3641 |
| **2007** | 5.3243 | 5.3859 | 14.2428 | 14.6900 | 30.3198 | 30.4693 | 58.9941 | 58.4014 | 90.3626 | 90.2868 | 109.4197 | 110.2806 | 129.6112 | 129.3608 |
| **2008** | 5.4869 | 5.4889 | 14.6012 | 14.9020 | 30.5910 | 30.8122 | 59.5082 | 58.8184 | 91.1905 | 90.6701 | 110.3347 | 111.4507 | 129.4532 | 129.6999 |
| **2009** | 5.6966 | 5.6020 | 15.0050 | 15.1346 | 30.8437 | 31.1954 | 59.3596 | 59.1966 | 92.1001 | 91.0398 | 111.0457 | 112.4526 | 128.3084 | 129.7736 |
| **2010** | 5.8370 | 5.6857 | 15.3285 | 15.3165 | 31.0366 | 31.4894 | 59.2870 | 59.5934 | 91.9344 | 90.7377 | 111.6701 | 112.9619 | 127.0802 | 129.4242 |
| **2011** | 5.8733 | 5.7488 | 15.5126 | 15.4627 | 31.1187 | 31.6628 | 59.4876 | 59.7988 | 90.8357 | 90.2967 | 111.6022 | 112.8190 | 127.0586 | 129.2945 |
| **2012** | 5.8272 | 5.7874 | 15.3938 | 15.5294 | 31.0761 | 31.6426 | 59.3166 | 59.6590 | 88.5234 | 89.4767 | 110.4013 | 111.9933 | 126.8590 | 128.7319 |
| **2013** | 5.9155 | 5.8498 | 15.5597 | 15.6951 | 31.5502 | 31.7953 | 59.8510 | 59.7618 | 87.8216 | 89.1846 | 110.1632 | 111.5639 | 127.0537 | 128.9729 |
| **2014** | 5.9601 | 5.9119 | 15.7191 | 15.9171 | 31.9289 | 32.0563 | 60.6784 | 60.1697 | 87.4272 | 89.2820 | 110.8541 | 111.2772 | 128.5011 | 129.3812 |
| **2015** | 5.9861 | 5.9987 | 15.8540 | 16.1353 | 32.4138 | 32.3866 | 61.0491 | 60.6933 | 87.0796 | 89.7141 | 111.7074 | 110.7006 | 130.4148 | 129.6043 |
| **2016** | 6.0504 | 6.0523 | 16.0140 | 16.2852 | 32.7463 | 32.6241 | 61.2456 | 60.7701 | 87.2477 | 89.7787 | 111.0034 | 109.7975 | 130.6362 | 129.1355 |
| **2017** | 6.1689 | 6.1296 | 16.2356 | 16.4360 | 33.0348 | 32.8322 | 61.9602 | 60.8988 | 87.7613 | 89.7854 | 110.8211 | 109.1449 | 130.3330 | 128.5711 |
| **2018** | 6.3362 | 6.2082 | 16.5343 | 16.6485 | 33.4743 | 33.3413 | 62.9404 | 61.4599 | 88.8635 | 90.3616 | 111.3516 | 109.2923 | 130.4413 | 128.5974 |
| **2019** | 6.4063 | 6.2449 | 16.6293 | 16.8448 | 33.8024 | 33.8354 | 63.2932 | 61.9892 | 89.5610 | 90.8813 | 111.7200 | 109.4241 | 130.4989 | 128.2929 |
| **2020** |  | 6.2483 |  | 17.0376 |  | 34.2253 |  | 62.4721 |  | 91.4630 |  | 109.6277 |  | 127.1922 |
| **2021** |  | 6.2343 |  | 17.2246 |  | 34.5359 |  | 62.9764 |  | 91.7377 |  | 109.8027 |  | 126.3571 |
| **2022** |  | 6.2488 |  | 17.4149 |  | 34.8170 |  | 63.5242 |  | 91.9450 |  | 109.7714 |  | 125.5981 |
| **2023** |  | 6.2495 |  | 17.6157 |  | 35.2348 |  | 64.2059 |  | 92.4519 |  | 110.1123 |  | 125.3458 |
| **2024** |  | 6.2410 |  | 17.7050 |  | 35.5830 |  | 65.0268 |  | 93.1219 |  | 110.8670 |  | 125.4015 |
| **2025** |  | 6.2456 |  | 17.7054 |  | 36.0057 |  | 65.8134 |  | 93.9767 |  | 111.3973 |  | 125.6797 |
| **2026** |  | 6.2469 |  | 17.7005 |  | 36.4510 |  | 66.4300 |  | 94.5854 |  | 111.7460 |  | 125.9841 |
| **2027** |  | 6.2502 |  | 17.6969 |  | 36.8669 |  | 67.0191 |  | 95.4338 |  | 112.0815 |  | 125.7847 |
| **2028** |  | 6.2501 |  | 17.7070 |  | 37.2111 |  | 67.7296 |  | 96.4538 |  | 112.5230 |  | 126.2318 |
| **2029** |  | 6.2533 |  | 17.7126 |  | 37.3602 |  | 68.4203 |  | 97.7415 |  | 113.5655 |  | 126.8907 |
| **2030** |  | 6.2440 |  | 17.6937 |  | 37.3939 |  | 69.1976 |  | 98.8089 |  | 114.3953 |  | 127.7208 |
| **2031** |  | 6.2360 |  | 17.6836 |  | 37.3653 |  | 69.9622 |  | 99.7427 |  | 115.3615 |  | 128.0419 |
| **2032** |  | 6.2413 |  | 17.6918 |  | 37.3529 |  | 70.8572 |  | 100.6961 |  | 116.2266 |  | 128.3637 |
| **2033** |  | 6.2385 |  | 17.7186 |  | 37.4067 |  | 71.4912 |  | 101.7176 |  | 117.4383 |  | 129.0147 |
| **2034** |  | 6.2535 |  | 17.6852 |  | 37.4033 |  | 71.9440 |  | 102.7868 |  | 119.0335 |  | 129.9955 |
| **2035** |  | 6.2387 |  | 17.6745 |  | 37.3482 |  | 71.8553 |  | 103.9740 |  | 120.3465 |  | 130.9045 |
| **2036** |  | 6.2401 |  | 17.6745 |  | 37.3491 |  | 71.8811 |  | 105.1591 |  | 121.5634 |  | 132.1531 |
| **2037** |  | 6.2402 |  | 17.6719 |  | 37.4064 |  | 71.8751 |  | 106.4438 |  | 122.6373 |  | 133.2573 |
| **2038** |  | 6.2403 |  | 17.6633 |  | 37.4056 |  | 71.8575 |  | 107.4455 |  | 123.9599 |  | 134.8466 |
| **2039** |  | 6.2482 |  | 17.6930 |  | 37.3683 |  | 71.8007 |  | 108.1326 |  | 125.2182 |  | 136.5478 |
| **2040** |  | 6.2413 |  | 17.6642 |  | 37.3687 |  | 71.9585 |  | 108.0144 |  | 126.6567 |  | 138.0520 |
| **2041** |  | 6.2400 |  | 17.6978 |  | 37.3450 |  | 71.7819 |  | 108.0075 |  | 128.1190 |  | 139.4299 |
| **2042** |  | 6.2452 |  | 17.6898 |  | 37.4143 |  | 71.7838 |  | 108.0002 |  | 129.7154 |  | 140.6923 |
| **2043** |  | 6.2456 |  | 17.6860 |  | 37.3743 |  | 71.8171 |  | 108.1920 |  | 130.8613 |  | 142.1549 |
| **2044** |  | 6.2562 |  | 17.6954 |  | 37.3583 |  | 71.9419 |  | 108.1527 |  | 131.5750 |  | 143.5679 |

## Incidence for FBC projections at the global level for 25-94 years at five-year age intervals, 2020-2044 (60-94 age group)

| **year** | **60-64** | | **65-69** | | **70-74** | | **75-79** | | **80-84** | | **85-89** | | **90-94** | |
| --- | --- | --- | --- | --- | --- | --- | --- | --- | --- | --- | --- | --- | --- | --- |
|  | **GBD value** | **Predicted value** | **GBD value** | **Predicted value** | **GBD value** | **Predicted value** | **GBD value** | **Predicted value** | **GBD value** | **Predicted value** | **GBD value** | **Predicted value** | **GBD value** | **Predicted value** |
| **1990** | 126.5984 | 126.0642 | 148.7494 | 150.0648 | 152.3995 | 153.5288 | 171.4228 | 171.1712 | 194.1463 | 195.5618 | 205.1919 | 201.0204 | 174.7923 | 173.3713 |
| **1991** | 127.0025 | 126.0635 | 149.3030 | 149.3983 | 155.7521 | 155.6270 | 173.1808 | 171.9508 | 196.5171 | 197.8448 | 206.7421 | 203.9216 | 174.3060 | 174.6791 |
| **1992** | 127.1154 | 126.5480 | 149.1358 | 149.1380 | 159.6226 | 158.8887 | 173.8939 | 172.6984 | 199.5933 | 200.6568 | 208.6170 | 207.2135 | 175.0420 | 176.5640 |
| **1993** | 129.3095 | 128.2430 | 151.5182 | 150.3370 | 165.4764 | 163.2612 | 177.1172 | 174.6767 | 205.5028 | 205.2985 | 215.5752 | 212.5482 | 179.2116 | 180.3443 |
| **1994** | 129.0995 | 129.5033 | 150.6955 | 150.4302 | 167.6817 | 165.5928 | 175.8083 | 175.4813 | 207.8534 | 208.2744 | 217.9263 | 216.4326 | 180.1668 | 183.3269 |
| **1995** | 128.6055 | 130.5518 | 149.7369 | 150.4676 | 167.6104 | 165.8957 | 176.5887 | 176.5958 | 209.2504 | 209.9877 | 221.8029 | 218.9960 | 182.7173 | 186.2255 |
| **1996** | 128.1857 | 131.0470 | 148.1254 | 149.8539 | 165.5934 | 164.7938 | 178.0770 | 178.5716 | 209.3854 | 209.8920 | 222.7763 | 220.8759 | 185.4818 | 188.1475 |
| **1997** | 128.8187 | 131.7373 | 147.0234 | 149.2303 | 163.5792 | 163.2885 | 179.8917 | 180.6750 | 208.2888 | 209.5226 | 223.2795 | 222.5501 | 186.9231 | 189.6737 |
| **1998** | 130.3039 | 133.7098 | 146.6739 | 149.2487 | 162.7525 | 162.3602 | 182.3806 | 183.4398 | 207.9572 | 209.2781 | 225.9370 | 224.6880 | 190.1946 | 192.0694 |
| **1999** | 133.1634 | 135.6622 | 147.2938 | 149.8073 | 162.1684 | 161.4280 | 185.4621 | 184.7775 | 207.4637 | 208.8701 | 229.5512 | 226.5921 | 194.0190 | 194.2813 |
| **2000** | 134.8322 | 137.0559 | 146.9529 | 150.3798 | 160.2152 | 160.6465 | 183.7760 | 184.2082 | 206.7246 | 208.9225 | 228.6016 | 226.8469 | 194.9282 | 195.7842 |
| **2001** | 136.5647 | 138.6186 | 147.3578 | 150.6207 | 159.1940 | 159.8958 | 181.3218 | 182.7239 | 207.7386 | 210.8539 | 227.1423 | 226.9813 | 195.8771 | 196.9052 |
| **2002** | 139.9494 | 139.9864 | 149.6144 | 151.4451 | 159.0706 | 158.5857 | 180.3779 | 180.2844 | 211.2705 | 213.1220 | 226.9925 | 225.9829 | 198.8587 | 197.8781 |
| **2003** | 143.4299 | 141.3534 | 152.5815 | 152.2403 | 159.0073 | 157.9268 | 179.3493 | 178.1700 | 213.8518 | 214.5726 | 224.2248 | 224.0473 | 200.1757 | 198.5550 |
| **2004** | 143.8837 | 141.9699 | 153.5658 | 152.5057 | 156.9203 | 156.3756 | 175.1330 | 175.2678 | 212.5093 | 213.7289 | 217.0724 | 220.5141 | 197.3346 | 197.6089 |
| **2005** | 145.4593 | 143.4064 | 155.6066 | 153.9895 | 157.0398 | 156.6353 | 174.3482 | 173.9983 | 211.5981 | 212.7391 | 217.0754 | 220.6133 | 198.2799 | 197.7477 |
| **2006** | 145.0309 | 143.7172 | 156.1865 | 154.7817 | 156.1412 | 156.2121 | 172.7516 | 172.0325 | 207.8351 | 209.7629 | 218.3003 | 221.3517 | 197.1997 | 196.4690 |
| **2007** | 146.9235 | 145.4230 | 158.6448 | 156.6994 | 156.8025 | 157.2754 | 172.6670 | 171.3228 | 206.2272 | 207.8226 | 222.8101 | 224.1806 | 196.7561 | 196.1934 |
| **2008** | 148.8678 | 147.5711 | 162.1622 | 159.5707 | 159.1370 | 159.4892 | 173.7480 | 171.9334 | 206.9676 | 207.0247 | 228.2789 | 227.9665 | 196.2171 | 196.1200 |
| **2009** | 149.6981 | 149.3546 | 165.0047 | 162.0476 | 161.3588 | 161.4726 | 173.7530 | 172.2088 | 205.7730 | 205.5737 | 230.5596 | 229.3425 | 193.9083 | 195.3494 |
| **2010** | 151.1740 | 150.8225 | 166.7810 | 163.9462 | 163.0261 | 163.2662 | 173.5073 | 172.5963 | 204.5184 | 204.4048 | 229.2004 | 228.5226 | 194.8452 | 195.6022 |
| **2011** | 151.6106 | 151.1909 | 167.3813 | 165.0660 | 164.6466 | 164.6866 | 173.7219 | 172.7183 | 204.7844 | 202.9888 | 227.1211 | 226.1008 | 199.5250 | 196.8385 |
| **2012** | 149.3309 | 150.0138 | 167.5105 | 165.6156 | 165.4711 | 165.5051 | 172.9218 | 172.4021 | 203.7507 | 200.3654 | 223.5884 | 222.1375 | 202.8624 | 197.8448 |
| **2013** | 148.0679 | 149.2056 | 167.3780 | 166.5595 | 166.9742 | 167.0526 | 173.4569 | 173.4431 | 202.8293 | 199.1833 | 220.4604 | 219.2707 | 204.4834 | 199.4318 |
| **2014** | 147.5334 | 148.4427 | 166.9681 | 167.3792 | 167.6615 | 168.5854 | 174.6611 | 174.7430 | 202.3516 | 198.3682 | 216.3601 | 216.5866 | 204.3595 | 199.6852 |
| **2015** | 147.4187 | 147.7523 | 169.0342 | 168.9449 | 169.5282 | 170.1924 | 176.7245 | 176.2818 | 202.7961 | 198.7586 | 215.4892 | 215.1046 | 204.0637 | 198.5911 |
| **2016** | 147.8045 | 147.2612 | 168.9700 | 168.7381 | 169.7519 | 170.8406 | 176.2635 | 177.2628 | 200.9339 | 198.2859 | 211.5191 | 212.8470 | 198.6218 | 195.8477 |
| **2017** | 148.8999 | 147.0947 | 167.6332 | 168.1171 | 170.7674 | 171.9634 | 175.2713 | 178.7560 | 199.9178 | 198.6270 | 205.5369 | 210.9865 | 191.2377 | 193.0084 |
| **2018** | 150.6807 | 147.7537 | 167.4022 | 167.6188 | 172.1792 | 173.7223 | 177.3745 | 180.8773 | 201.8902 | 200.3955 | 203.8165 | 210.2576 | 187.8618 | 191.1102 |
| **2019** | 151.3231 | 148.3061 | 167.8017 | 166.7246 | 172.7349 | 174.7955 | 179.4100 | 182.7299 | 202.1340 | 201.8938 | 203.7308 | 209.7325 | 184.8989 | 188.9122 |
| **2020** |  | 148.1143 |  | 165.6748 |  | 175.7117 |  | 184.1555 |  | 203.3627 |  | 209.3233 |  | 187.2733 |
| **2021** |  | 147.7345 |  | 165.2422 |  | 175.6262 |  | 184.9366 |  | 204.7660 |  | 209.0439 |  | 185.3342 |
| **2022** |  | 147.2881 |  | 165.1092 |  | 174.9363 |  | 186.3183 |  | 206.2226 |  | 209.4715 |  | 183.7766 |
| **2023** |  | 146.6963 |  | 165.5178 |  | 174.1782 |  | 187.3421 |  | 208.3604 |  | 210.6500 |  | 182.8216 |
| **2024** |  | 146.3515 |  | 165.8977 |  | 172.9664 |  | 188.4252 |  | 210.0208 |  | 212.0961 |  | 181.8181 |
| **2025** |  | 144.9458 |  | 165.8178 |  | 171.8916 |  | 189.3797 |  | 211.4738 |  | 213.4849 |  | 181.7655 |
| **2026** |  | 143.9802 |  | 165.2580 |  | 171.4208 |  | 189.4945 |  | 212.4826 |  | 214.7571 |  | 181.3610 |
| **2027** |  | 143.1290 |  | 164.5653 |  | 171.3287 |  | 188.3633 |  | 214.0746 |  | 216.7010 |  | 181.6145 |
| **2028** |  | 143.0123 |  | 164.1826 |  | 171.6622 |  | 187.8850 |  | 215.5358 |  | 218.7289 |  | 182.7629 |
| **2029** |  | 142.8900 |  | 163.4324 |  | 171.9324 |  | 186.6428 |  | 216.5065 |  | 220.6261 |  | 183.9384 |
| **2030** |  | 143.3246 |  | 162.2106 |  | 171.9175 |  | 185.3581 |  | 217.5703 |  | 222.2880 |  | 184.9192 |
| **2031** |  | 143.3980 |  | 160.9525 |  | 171.5353 |  | 184.6941 |  | 217.6810 |  | 223.2552 |  | 186.3378 |
| **2032** |  | 143.4741 |  | 160.0693 |  | 170.7937 |  | 184.4329 |  | 216.6878 |  | 224.7025 |  | 188.0561 |
| **2033** |  | 143.8824 |  | 159.6092 |  | 170.1845 |  | 185.0266 |  | 215.5781 |  | 226.5350 |  | 189.6613 |
| **2034** |  | 144.5633 |  | 159.5758 |  | 169.2698 |  | 185.2609 |  | 214.1155 |  | 227.3743 |  | 191.2769 |
| **2035** |  | 145.4344 |  | 159.9109 |  | 167.9298 |  | 185.0440 |  | 212.5723 |  | 228.4568 |  | 192.6589 |
| **2036** |  | 146.0027 |  | 160.2388 |  | 166.9579 |  | 184.6437 |  | 212.2319 |  | 228.7648 |  | 193.5703 |
| **2037** |  | 146.3390 |  | 160.3544 |  | 165.7001 |  | 183.8983 |  | 211.8066 |  | 227.8318 |  | 194.9047 |
| **2038** |  | 147.0310 |  | 161.0281 |  | 165.5147 |  | 183.1076 |  | 212.2511 |  | 226.2795 |  | 196.1806 |
| **2039** |  | 147.9803 |  | 161.8497 |  | 165.5162 |  | 182.8386 |  | 213.0360 |  | 224.9877 |  | 197.0959 |
| **2040** |  | 149.3697 |  | 162.6106 |  | 166.0806 |  | 181.0599 |  | 212.7158 |  | 223.6778 |  | 198.3102 |
| **2041** |  | 150.7514 |  | 163.0743 |  | 166.3669 |  | 180.0359 |  | 212.4437 |  | 222.9191 |  | 198.2249 |
| **2042** |  | 151.8208 |  | 163.5326 |  | 166.3705 |  | 179.2121 |  | 211.3638 |  | 222.8051 |  | 197.4683 |
| **2043** |  | 153.4653 |  | 164.4940 |  | 166.9380 |  | 178.4996 |  | 210.7693 |  | 223.0127 |  | 196.4171 |
| **2044** |  | 155.5248 |  | 165.6904 |  | 167.7309 |  | 178.6400 |  | 210.0799 |  | 223.4441 |  | 195.2365 |

# supplemental file5

The ASIR of FBC for 204 countries worldwide from 2019-2044

## Central Europe, eastern Europe, and central Asia (29 countries)


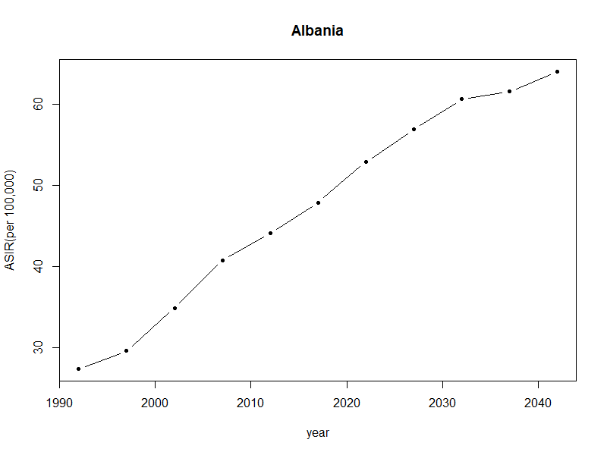

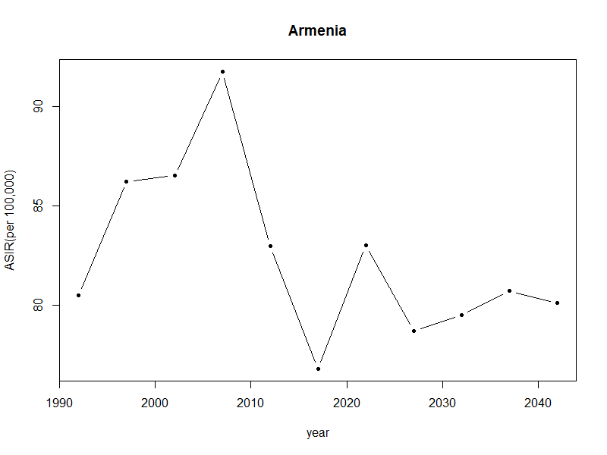


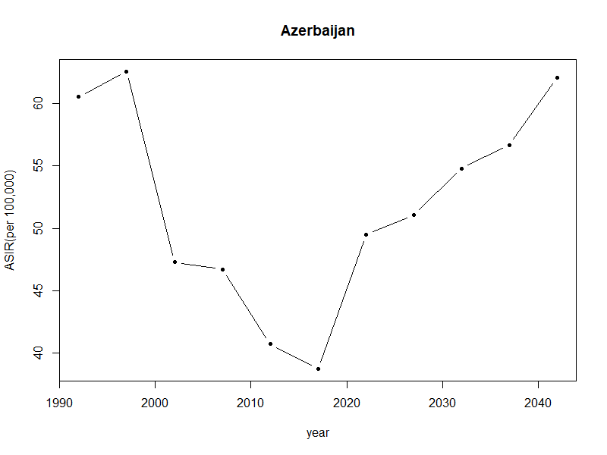

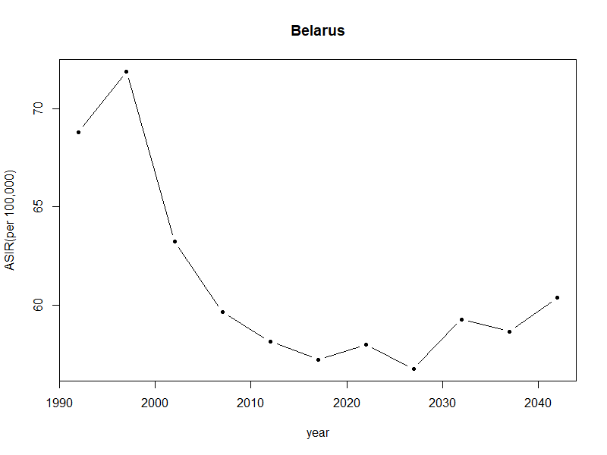


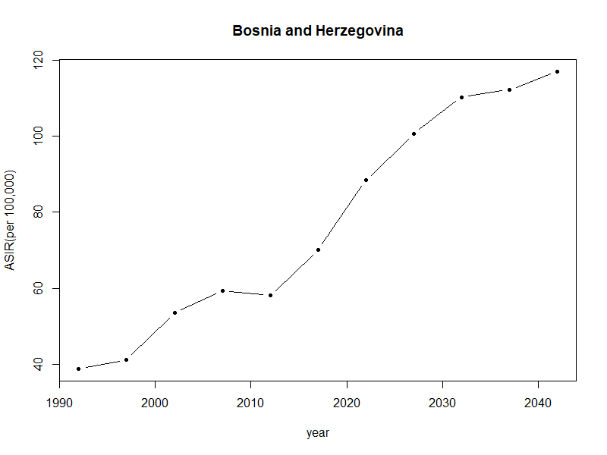

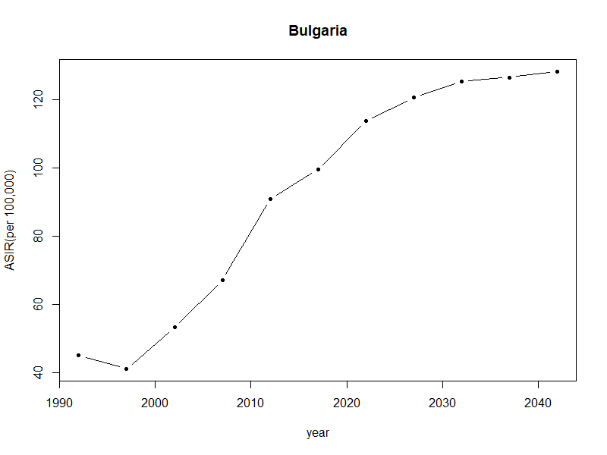


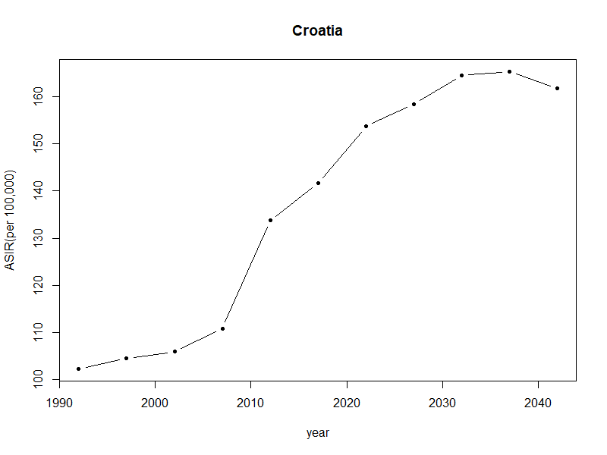

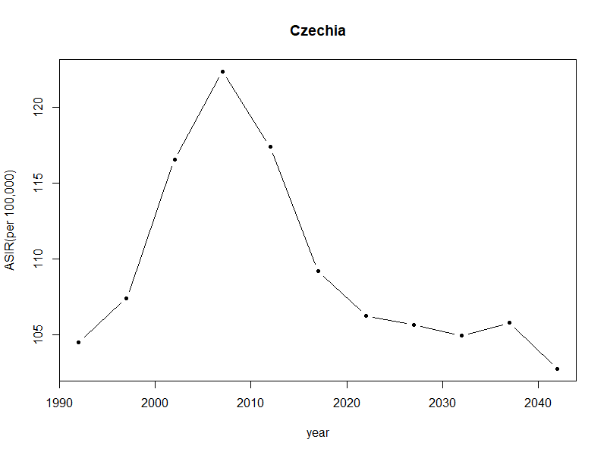


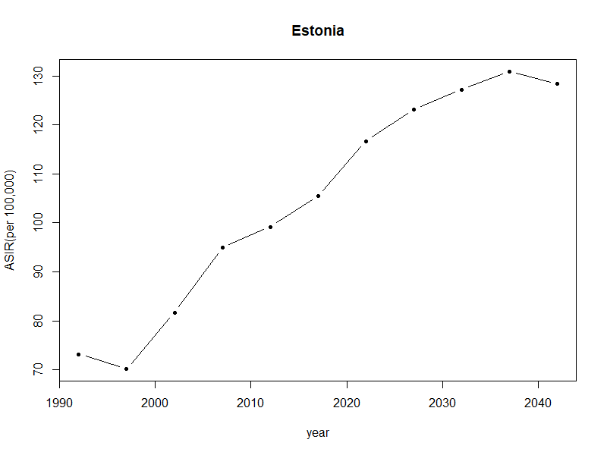

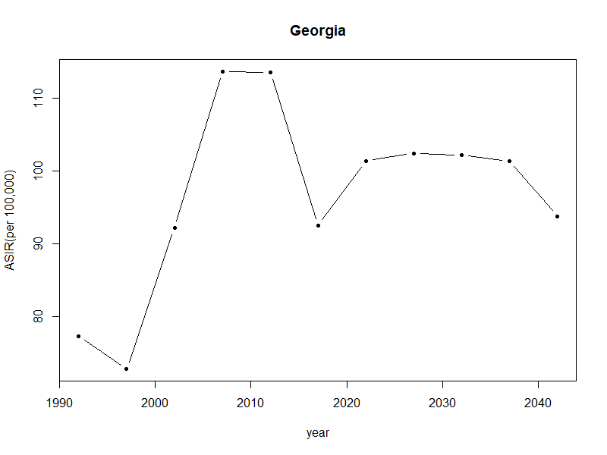


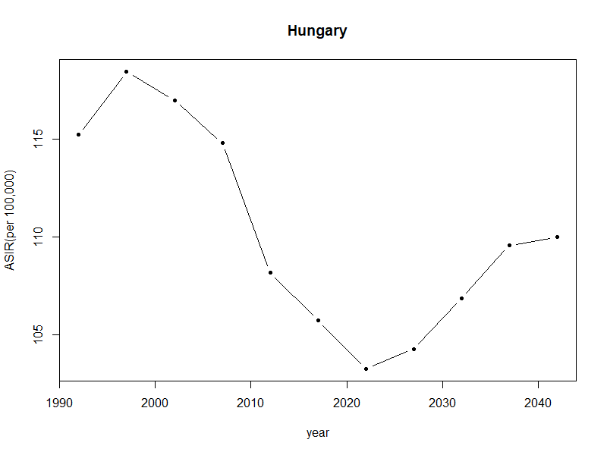

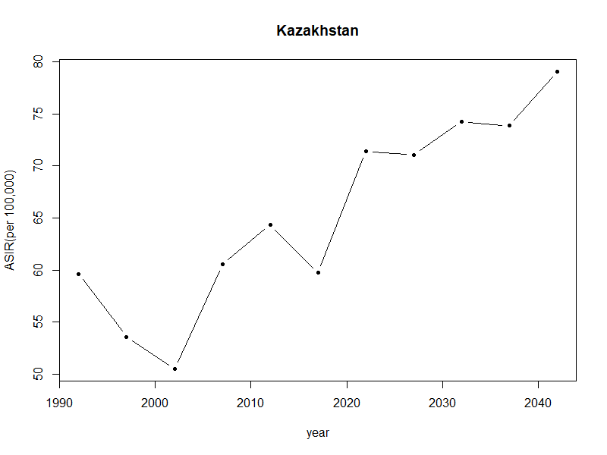


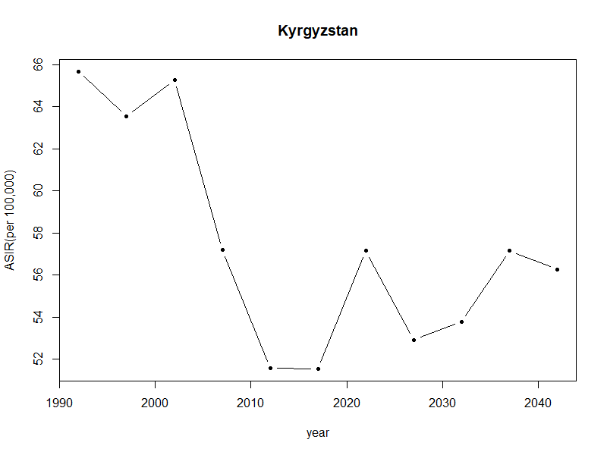

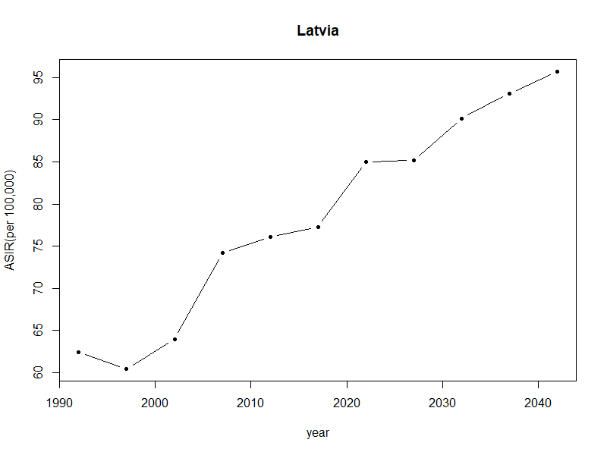


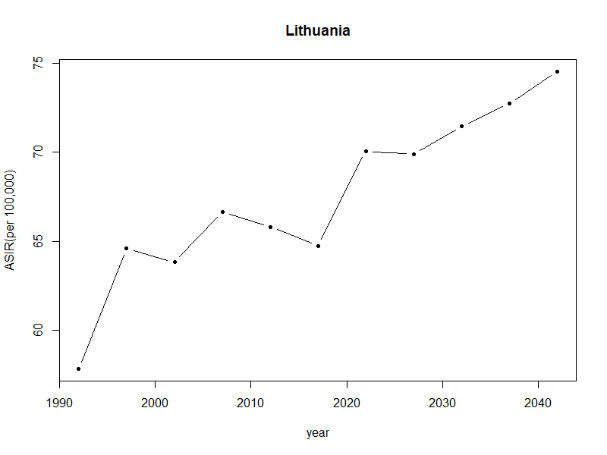

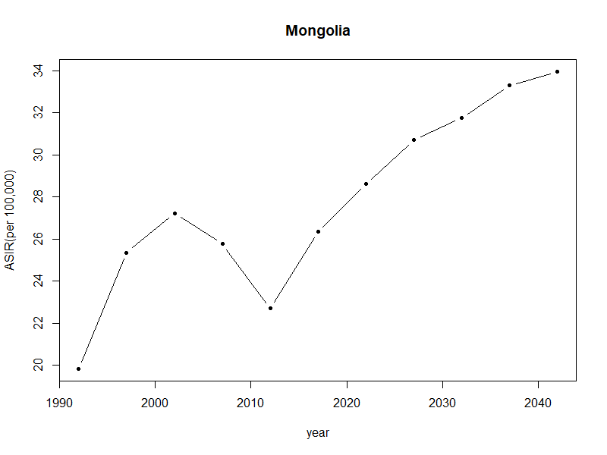


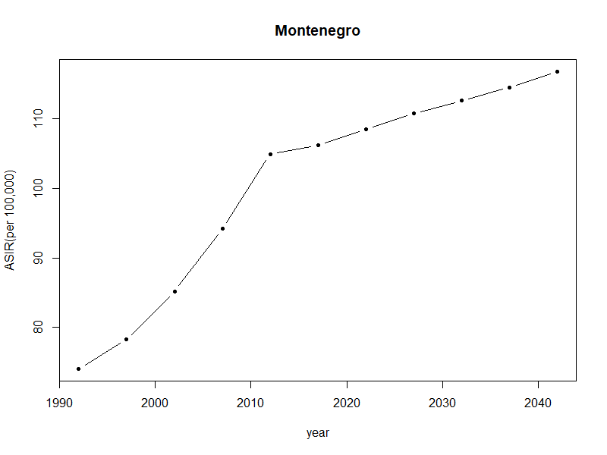

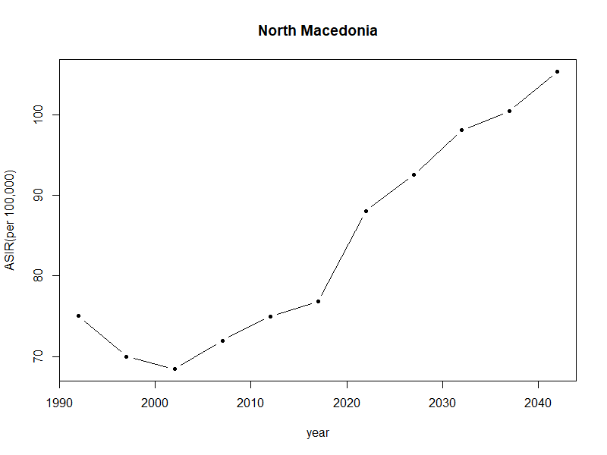


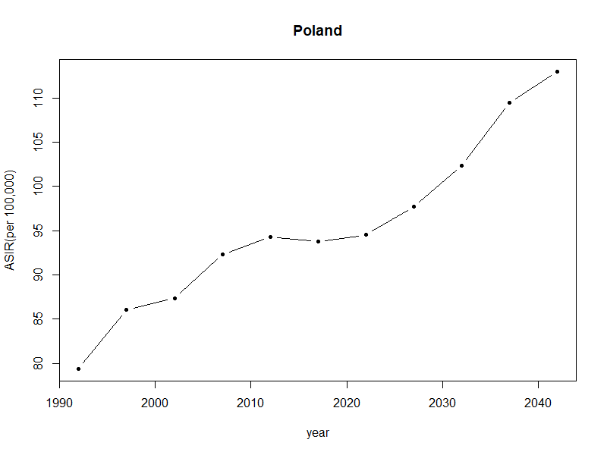

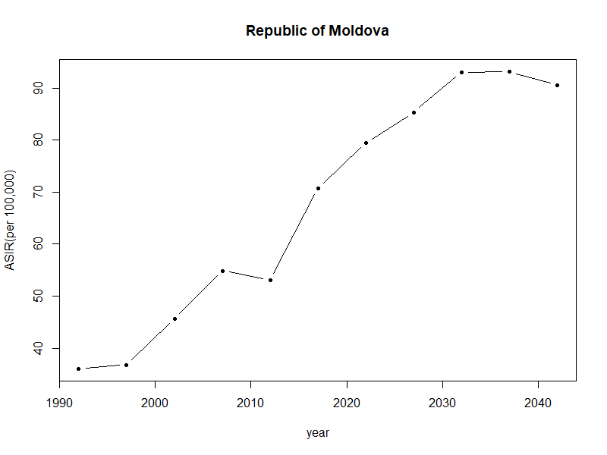


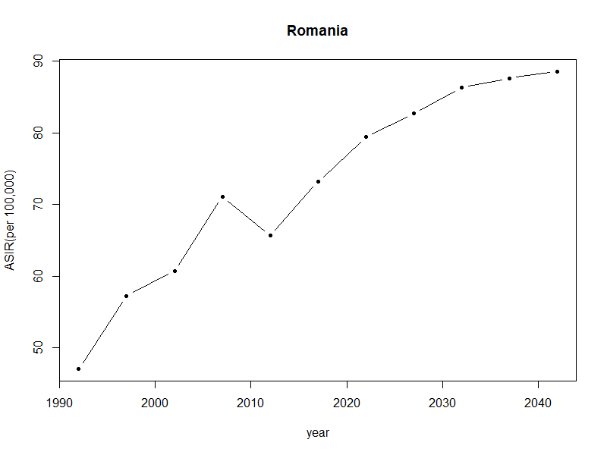

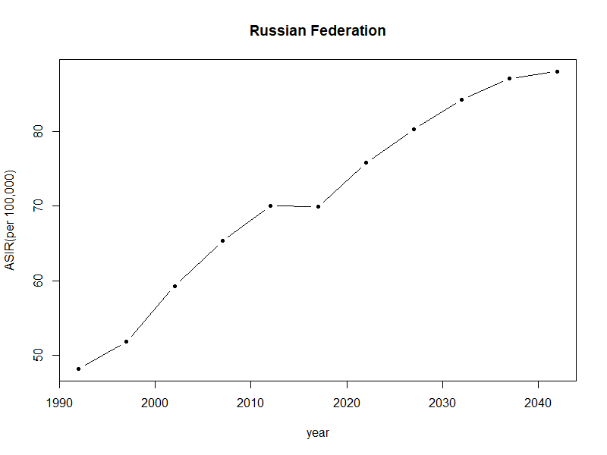


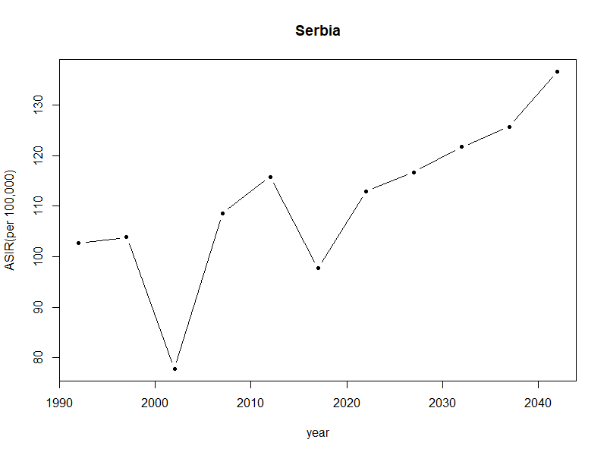

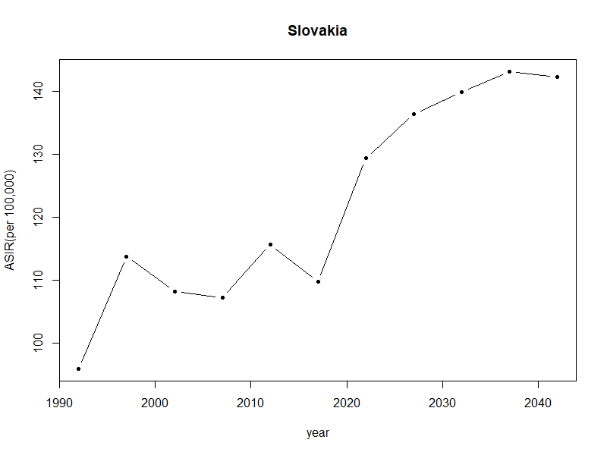


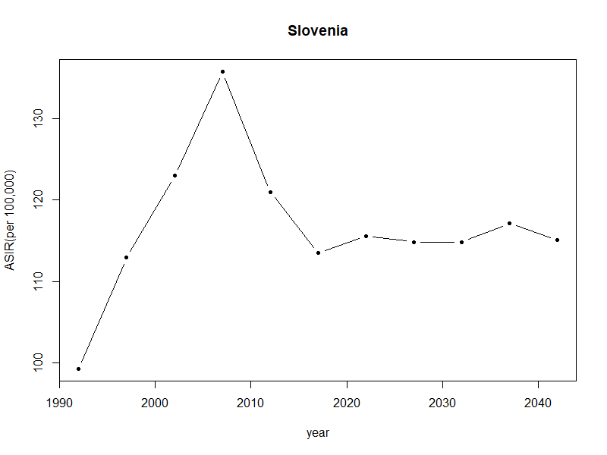

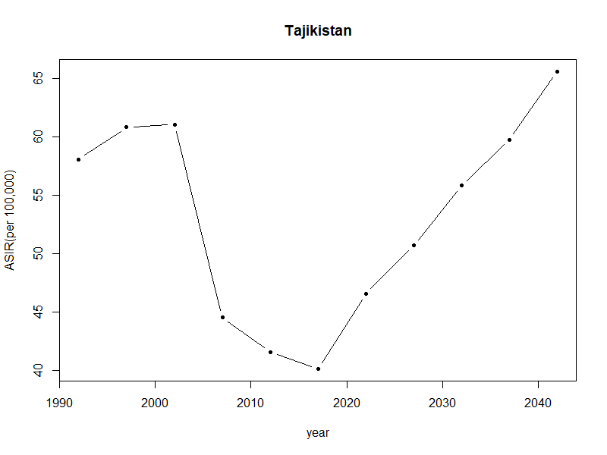


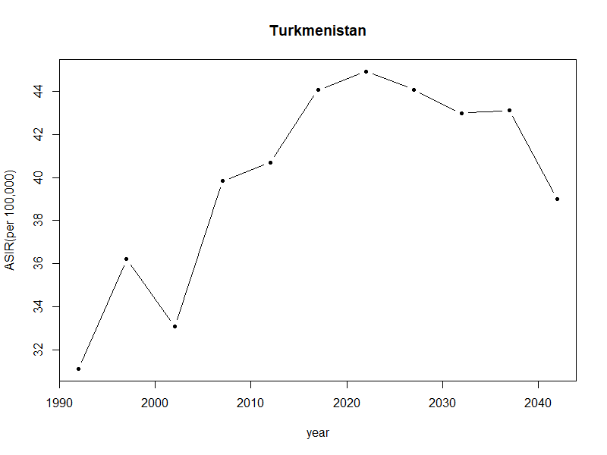

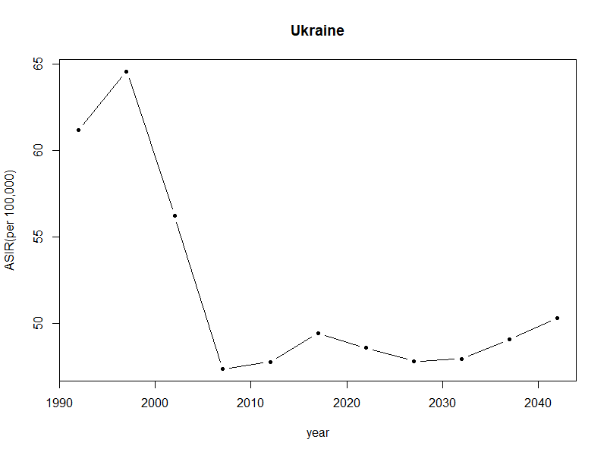


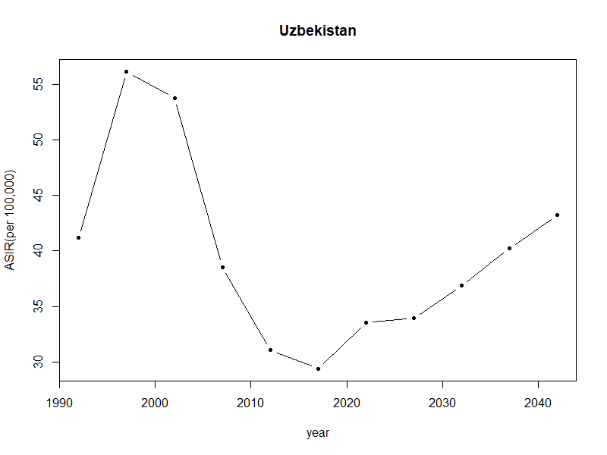


## High income (36 countries)


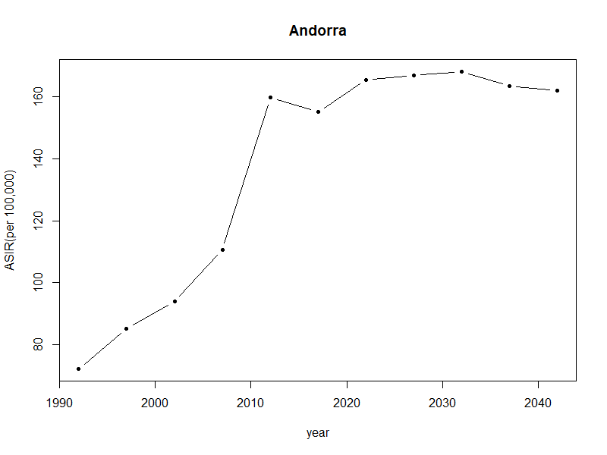

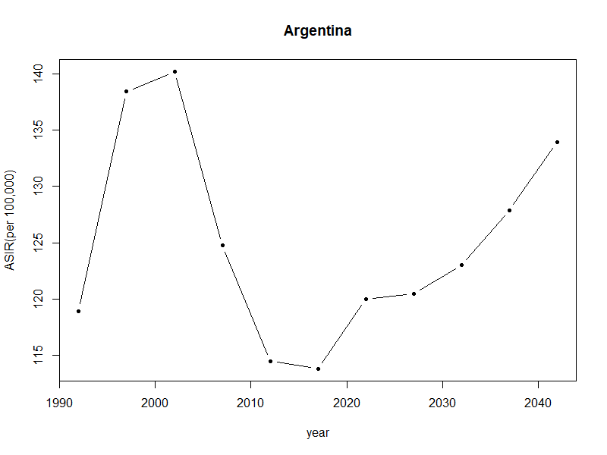


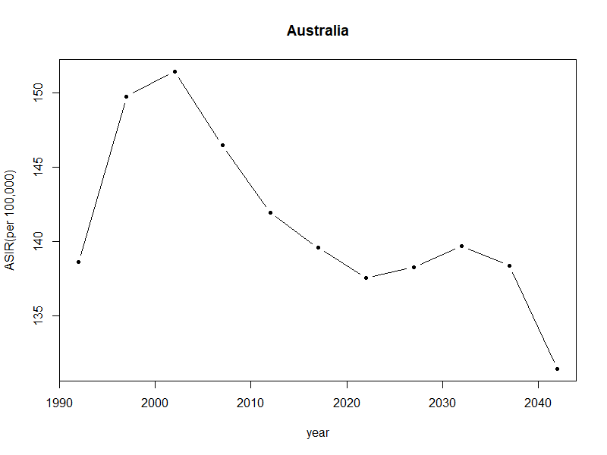

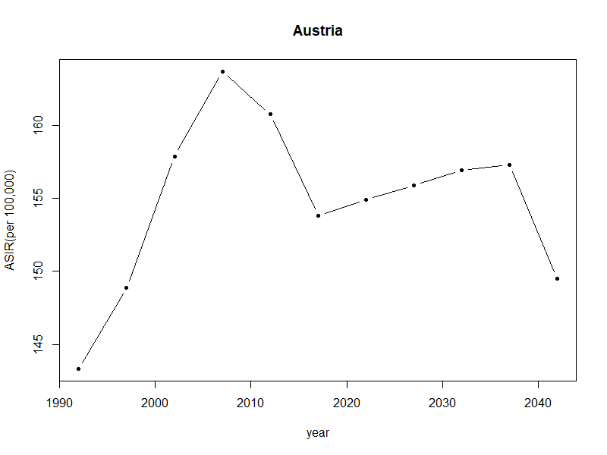


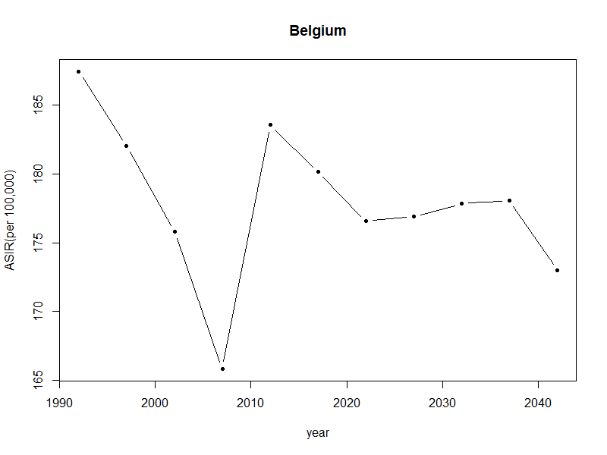

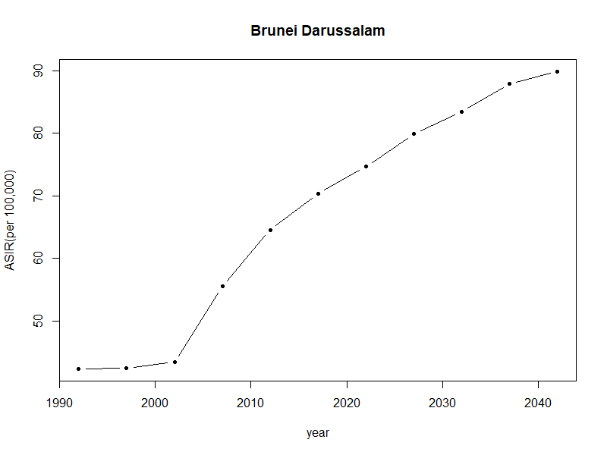


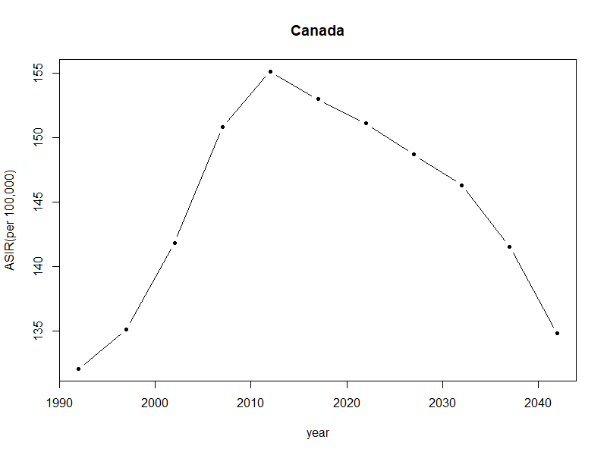

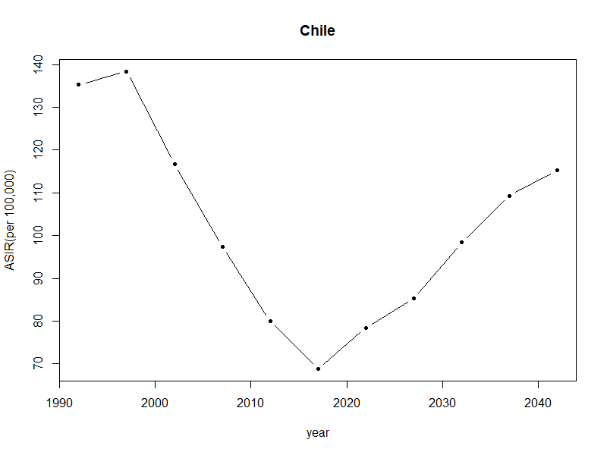


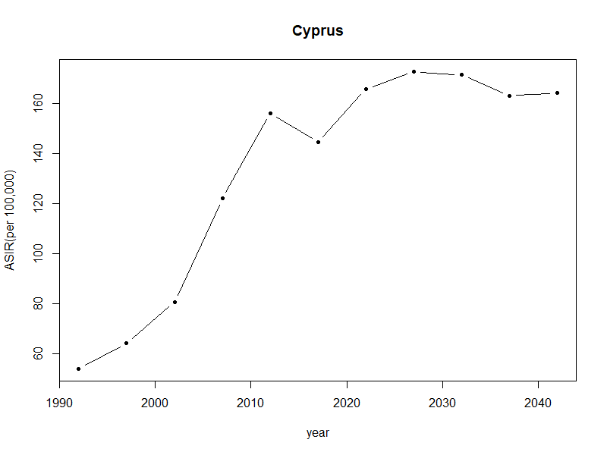

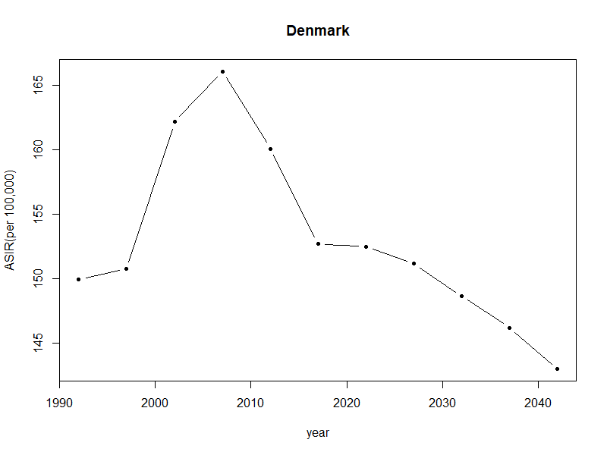


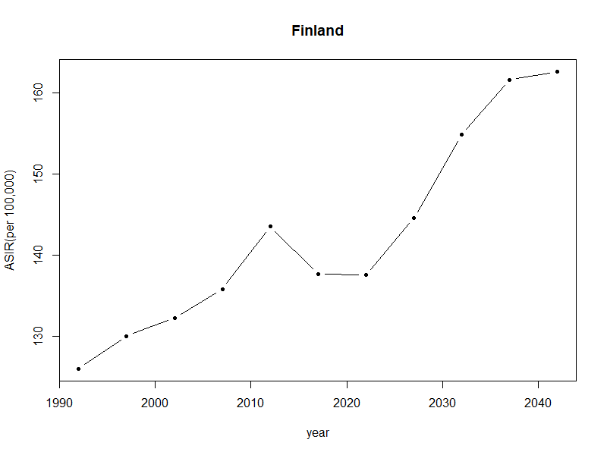

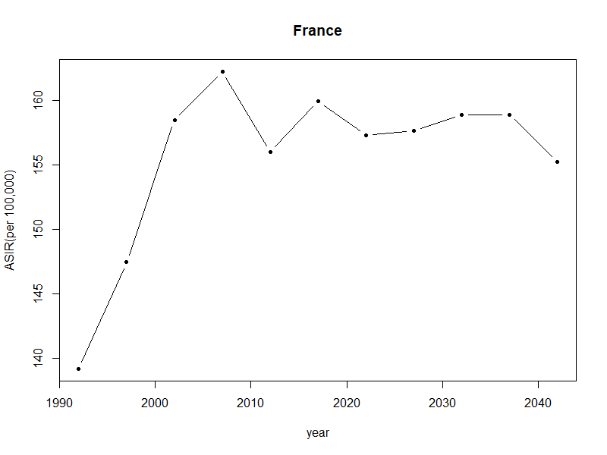


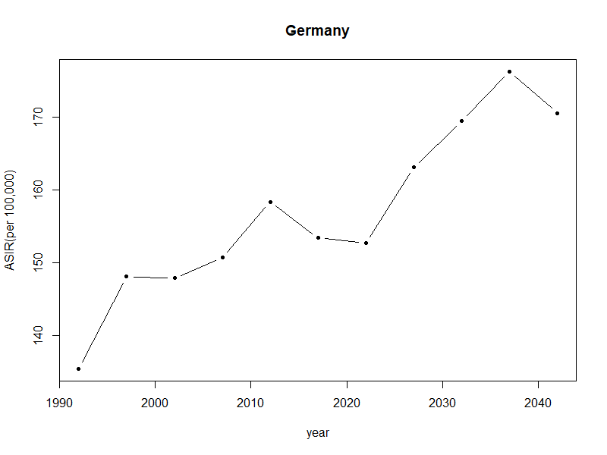

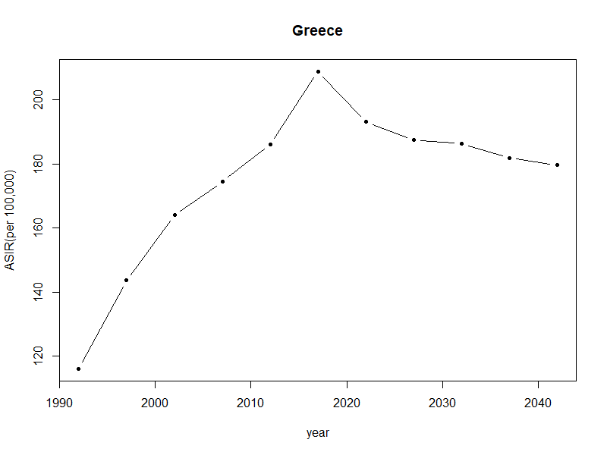


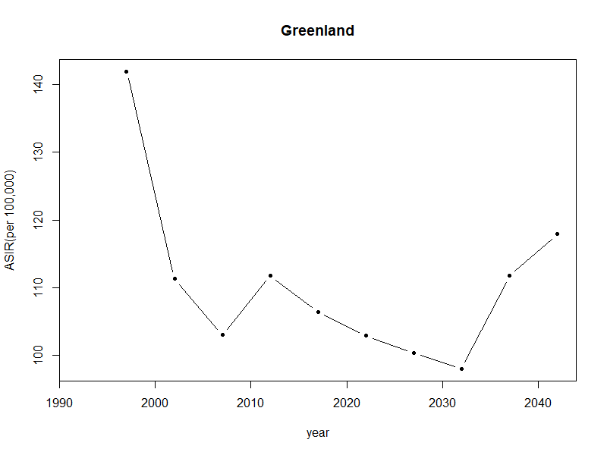

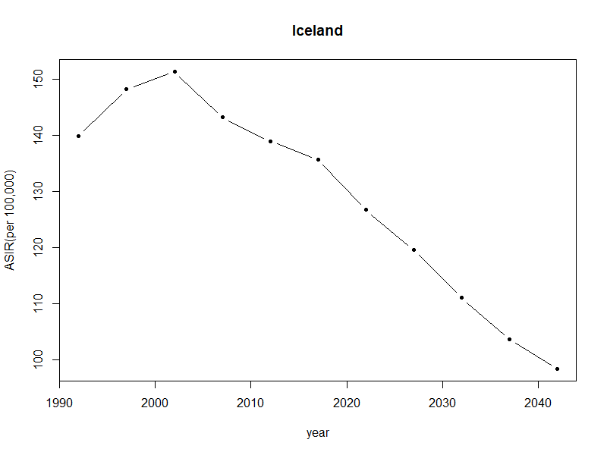


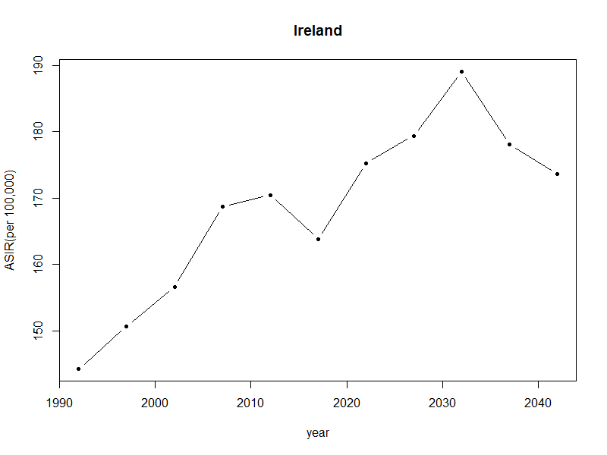

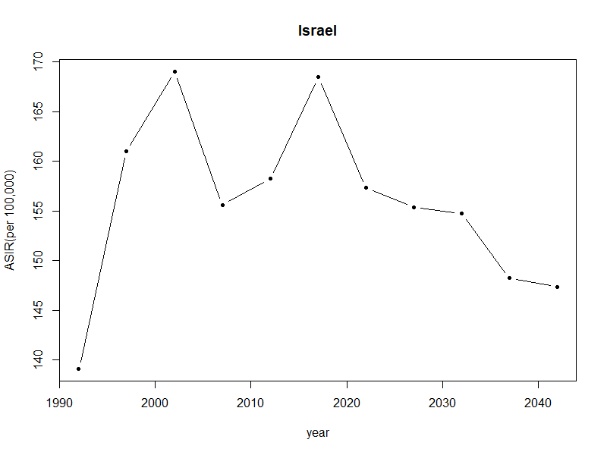


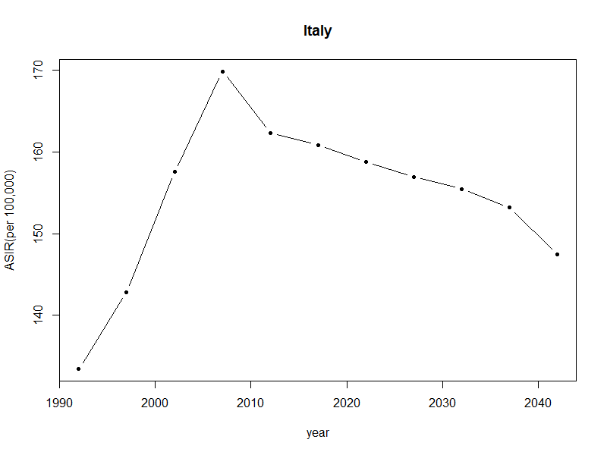

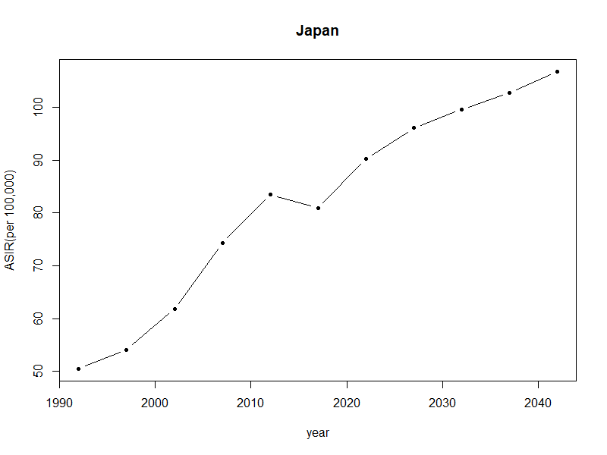


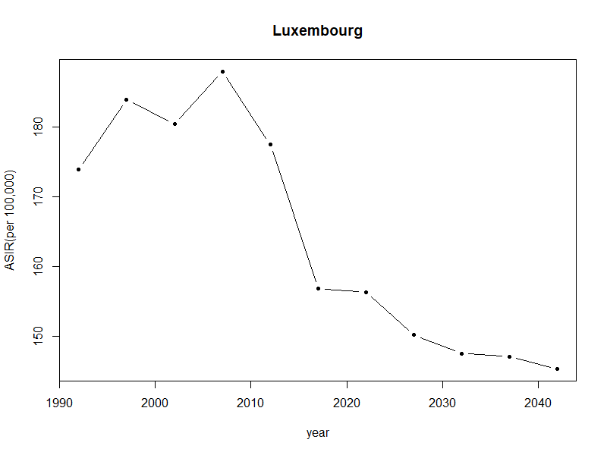

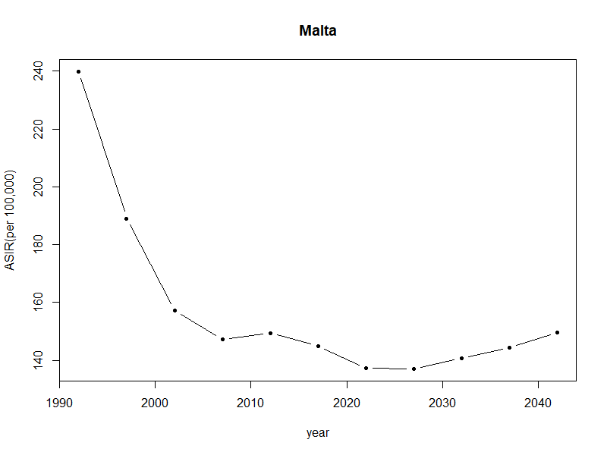


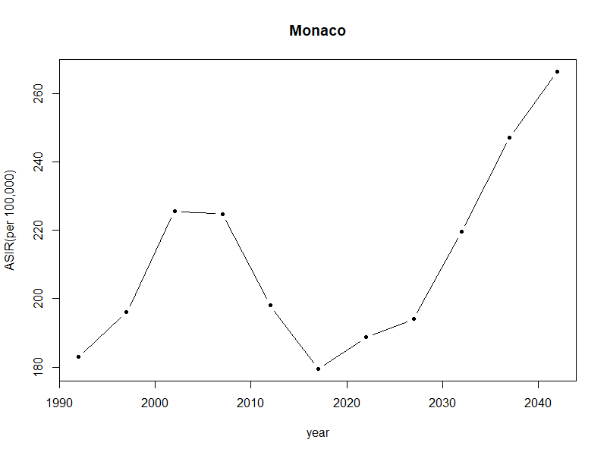

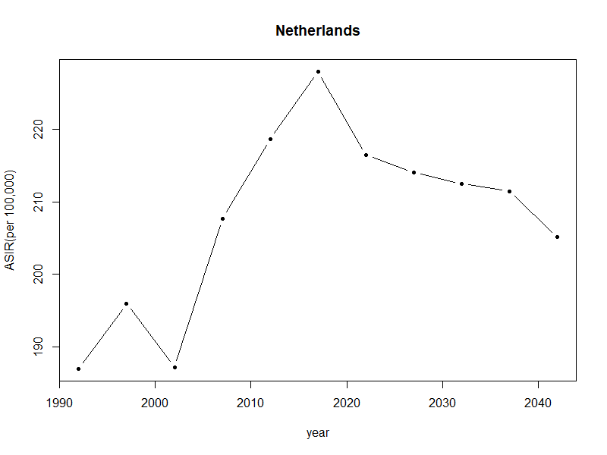


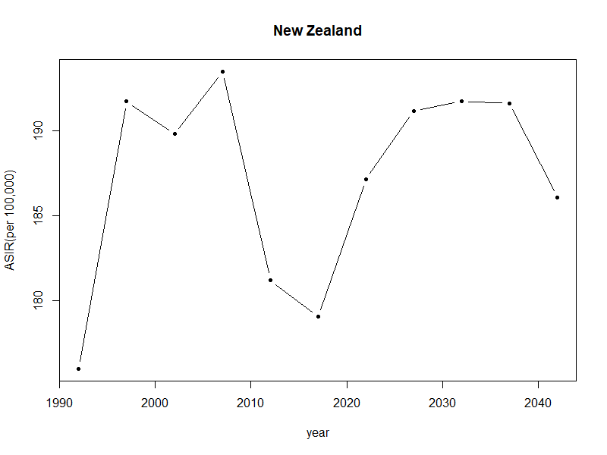

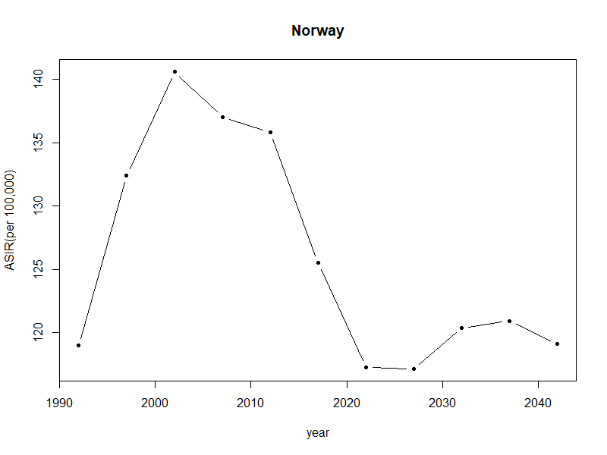


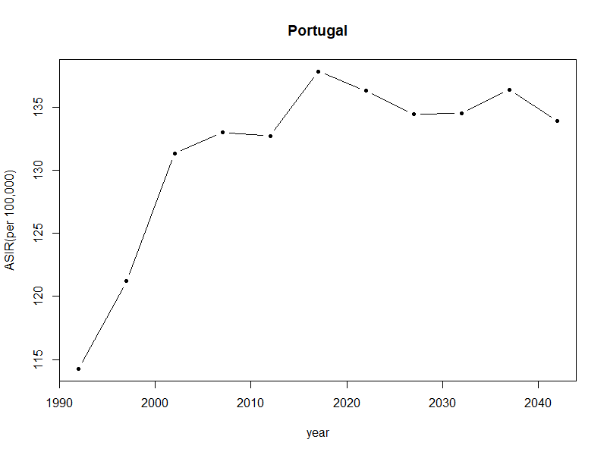

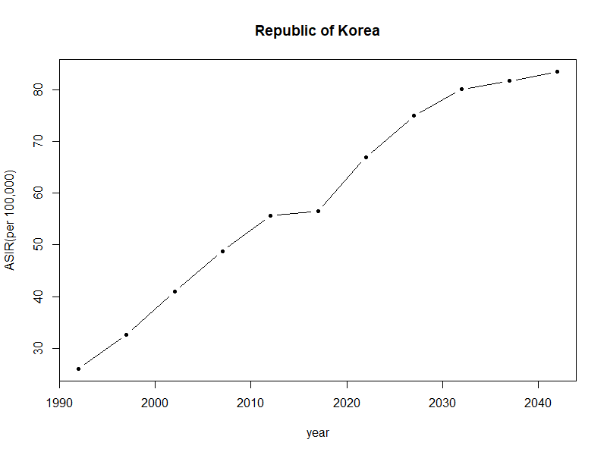


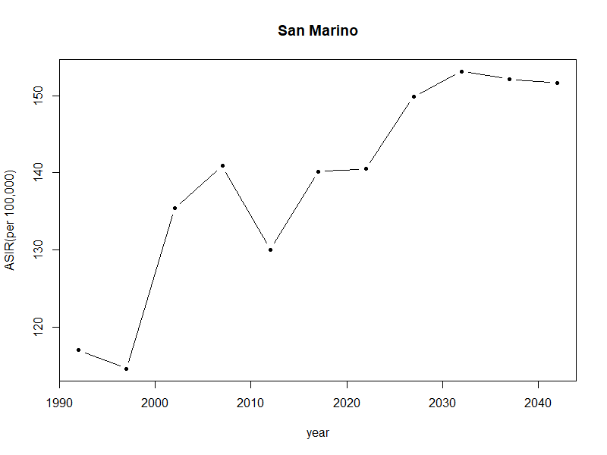

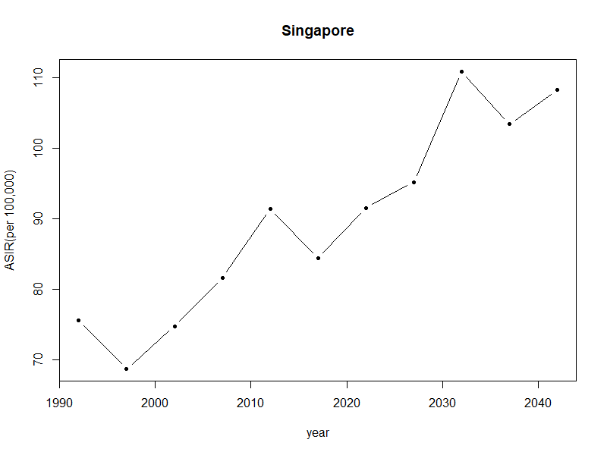


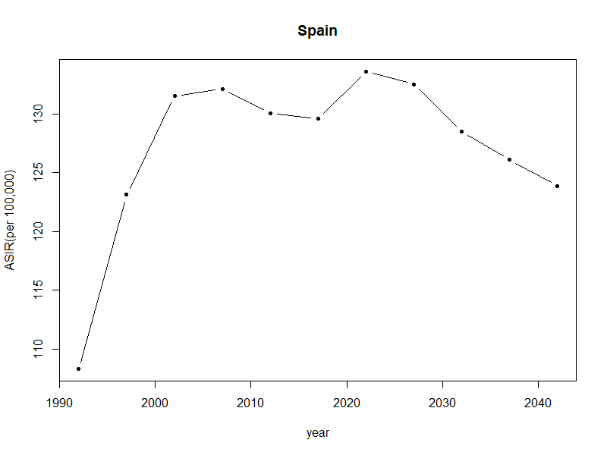

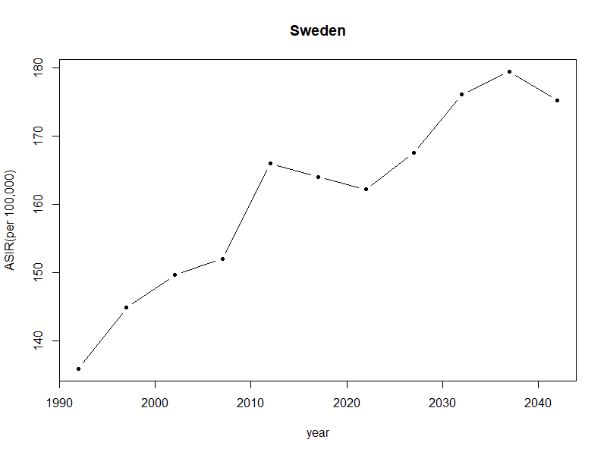


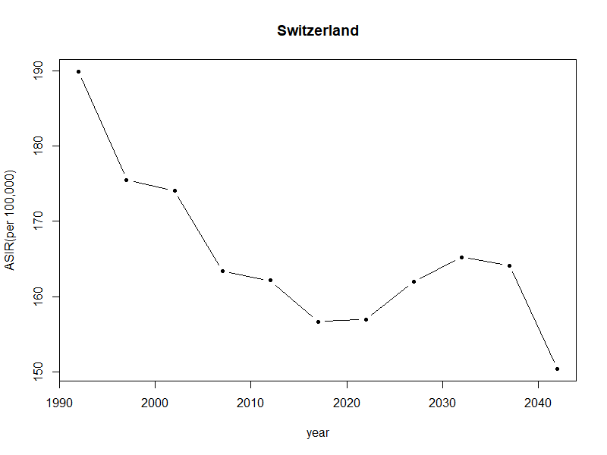

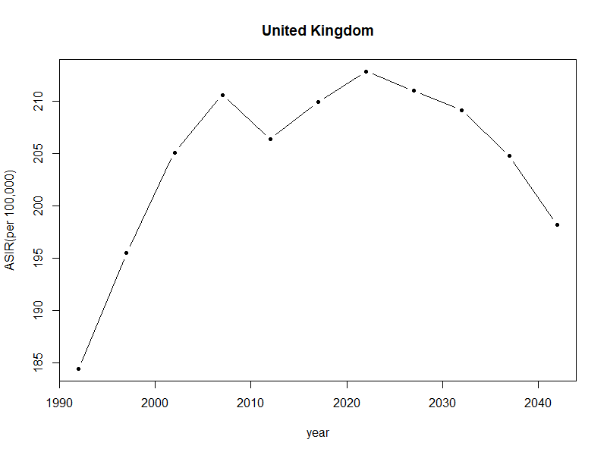


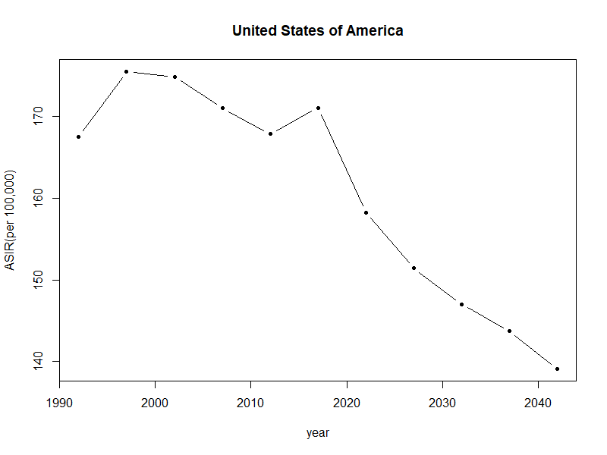

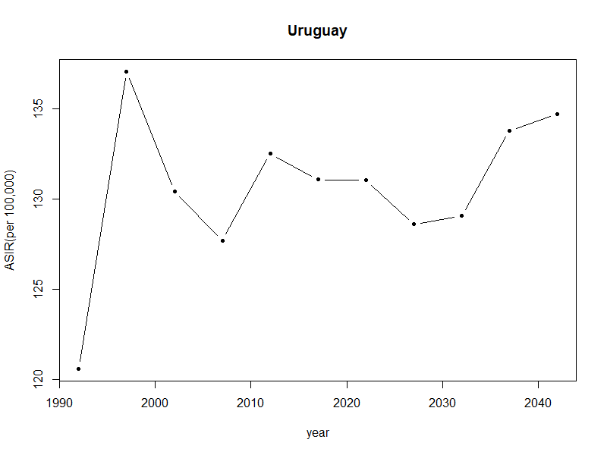


## Latin America and Caribbean (33 countries)


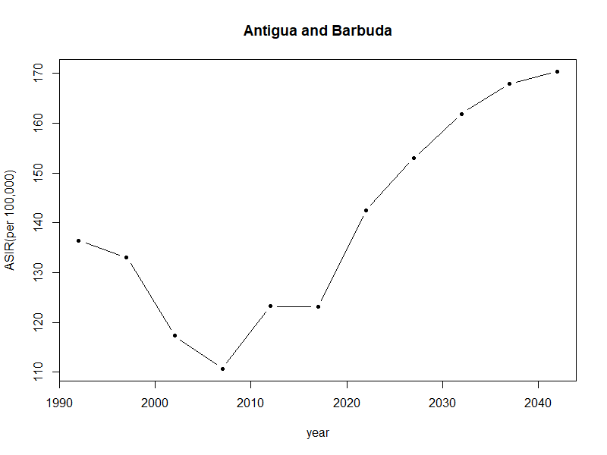

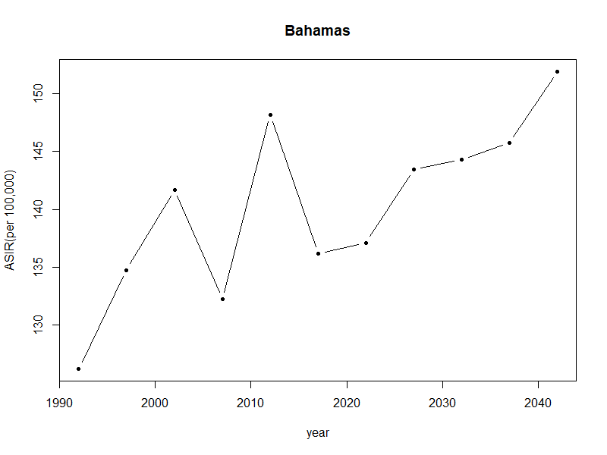


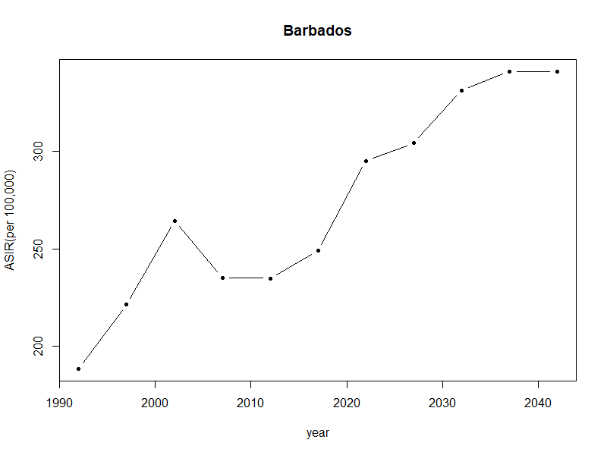

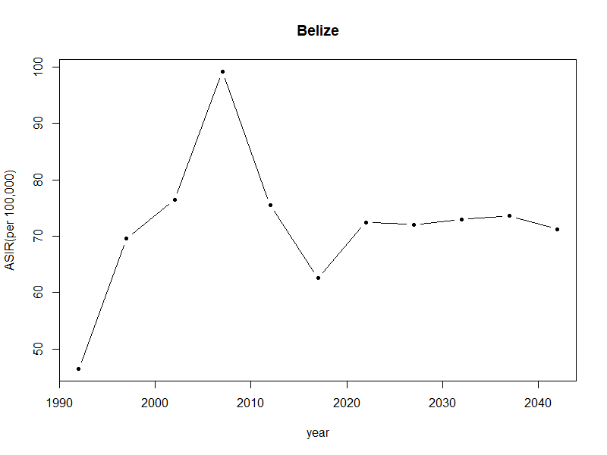


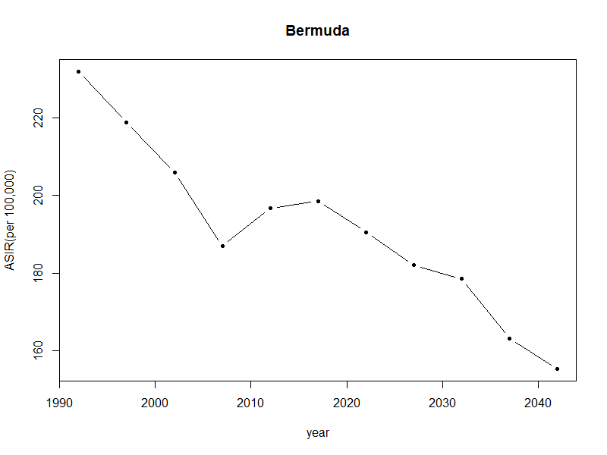

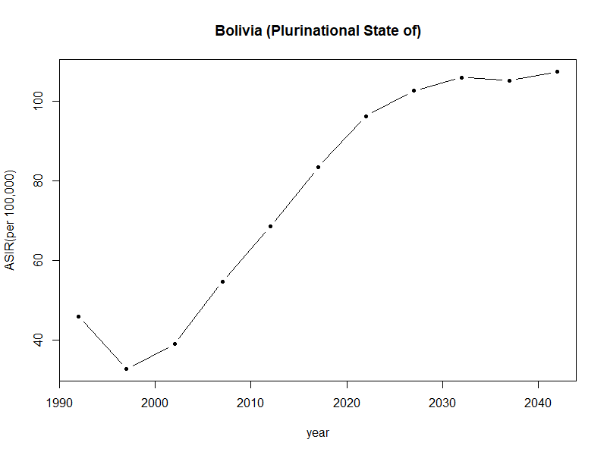


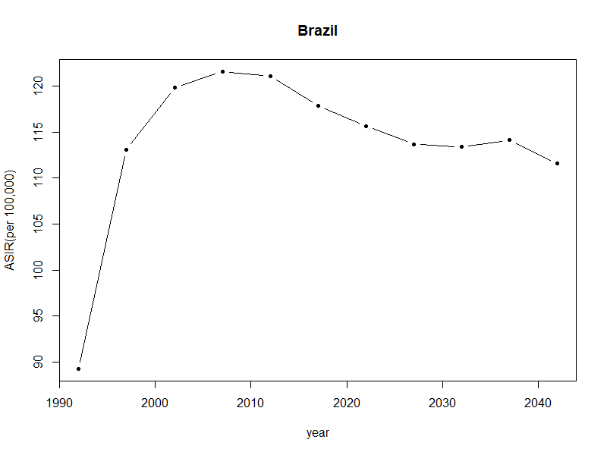

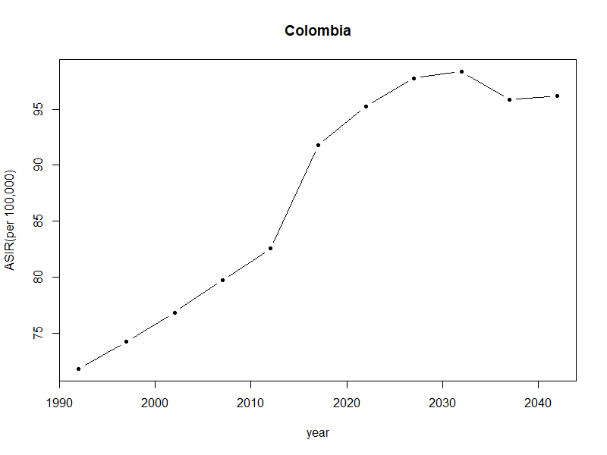


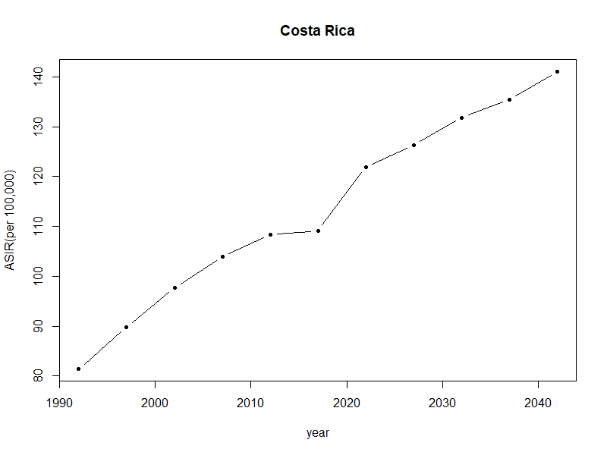

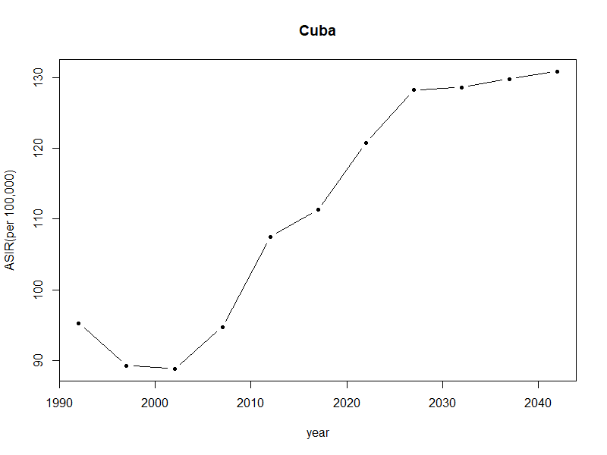


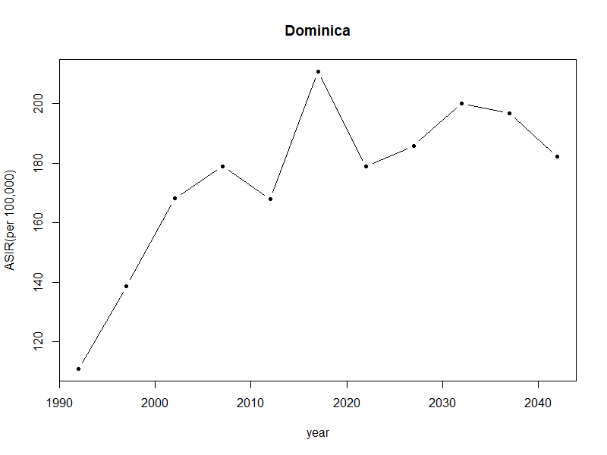

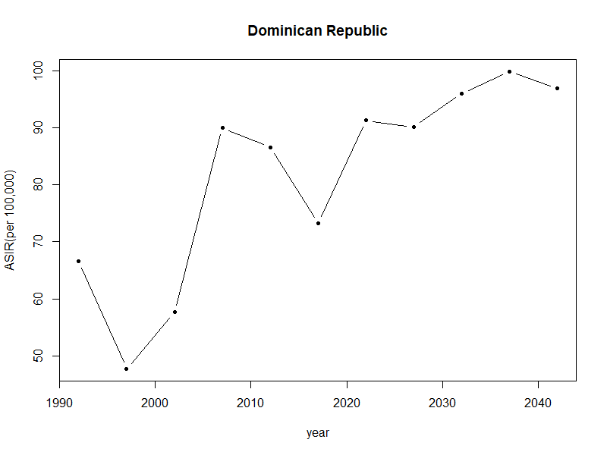


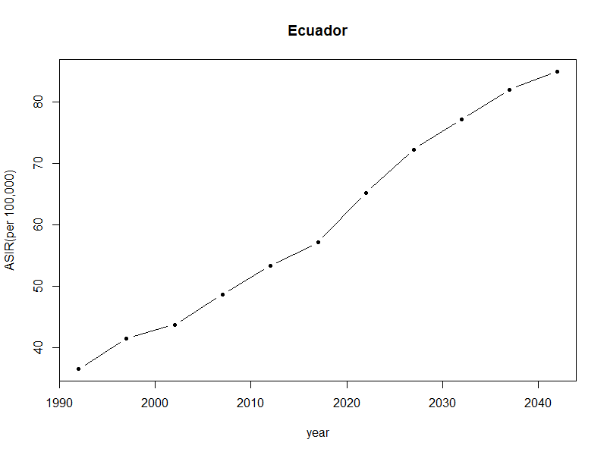

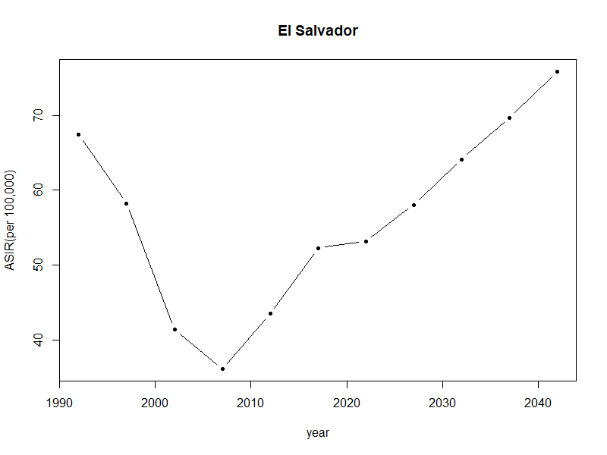


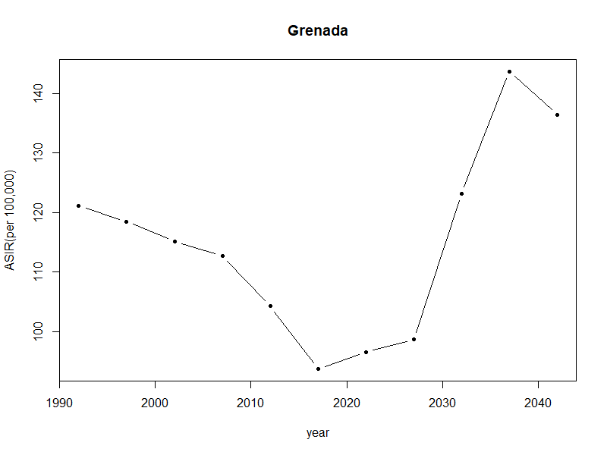

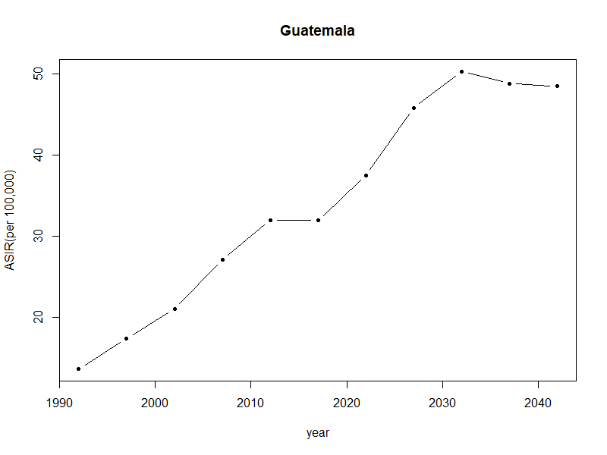


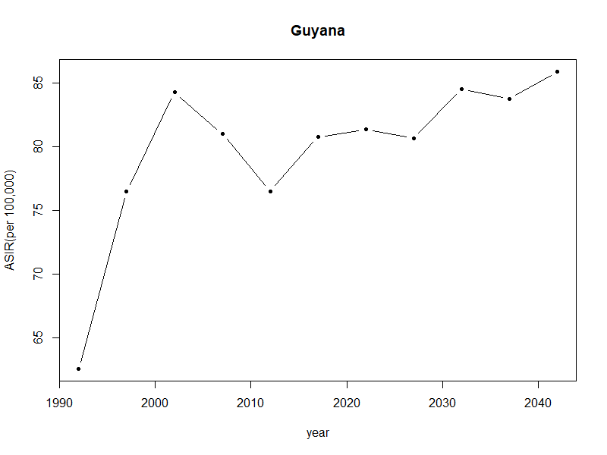

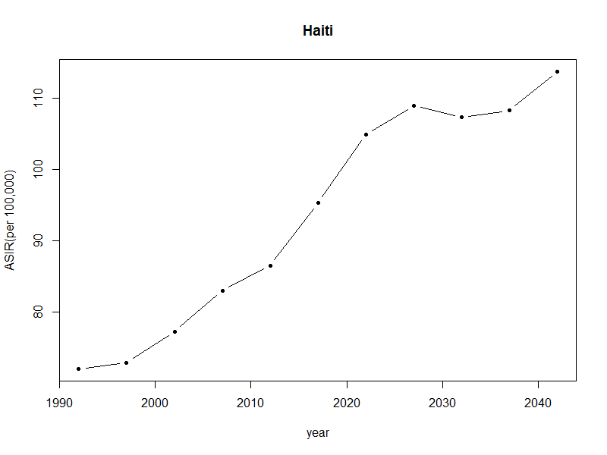


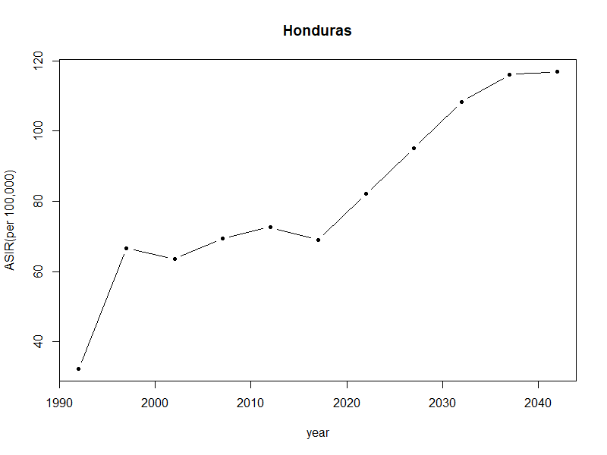

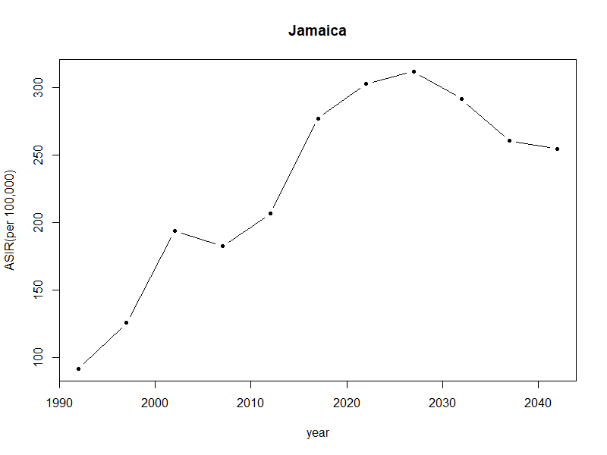


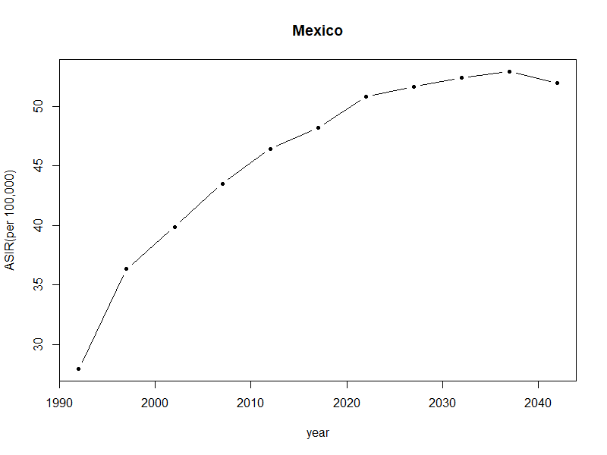

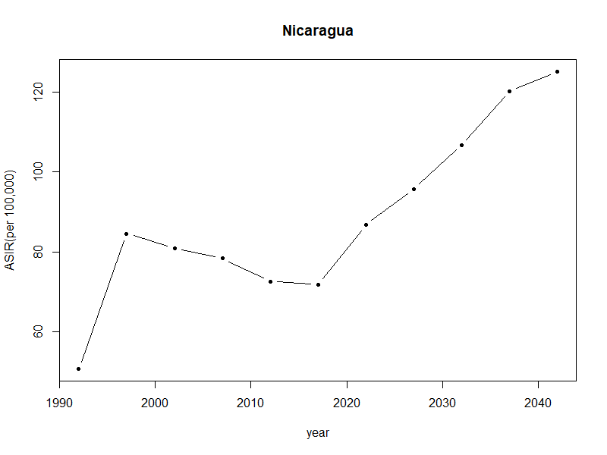


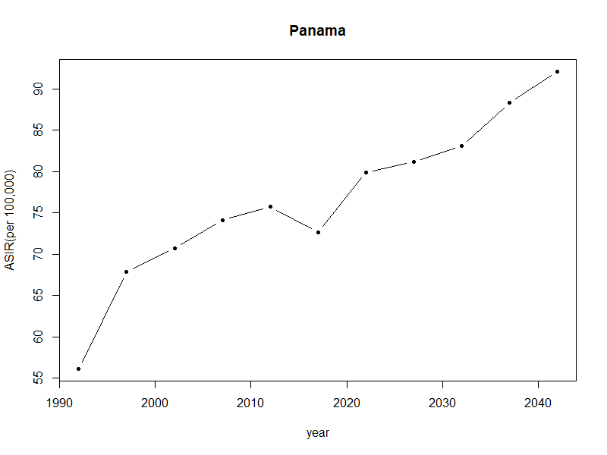

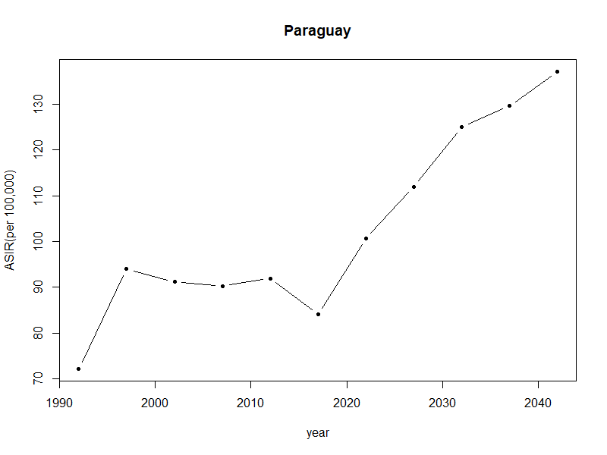


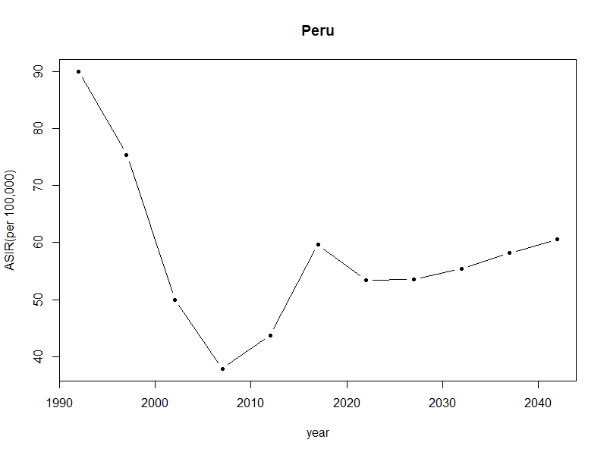

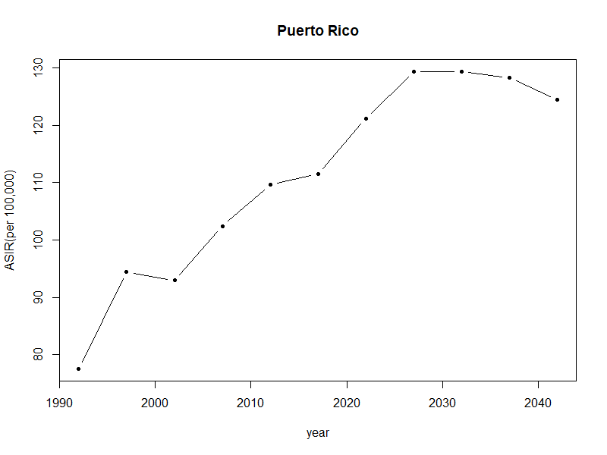


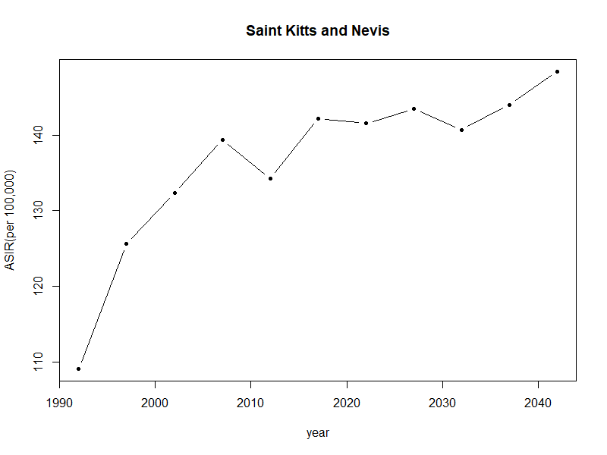

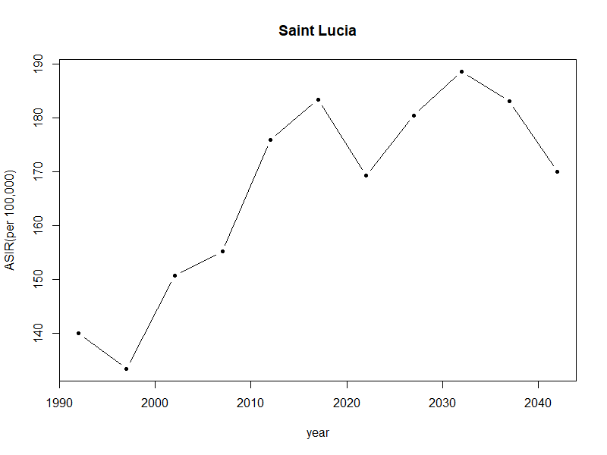


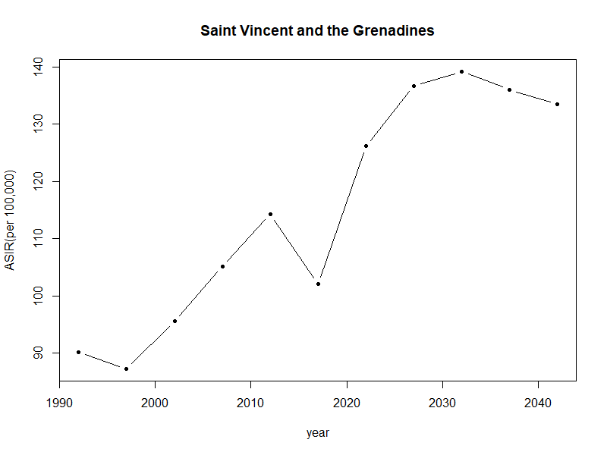

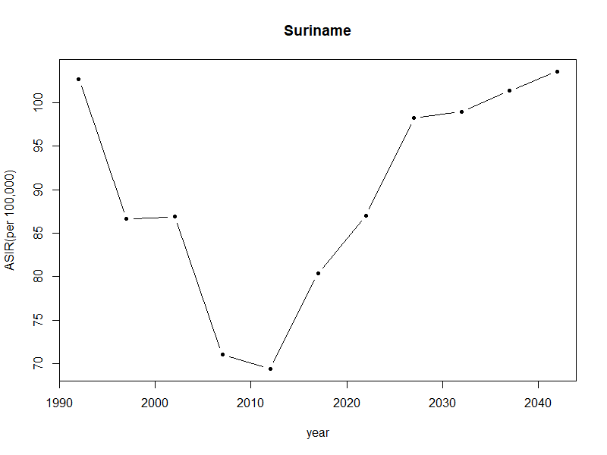


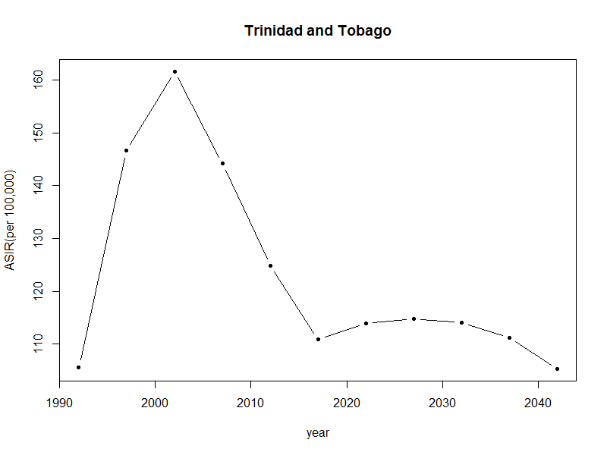

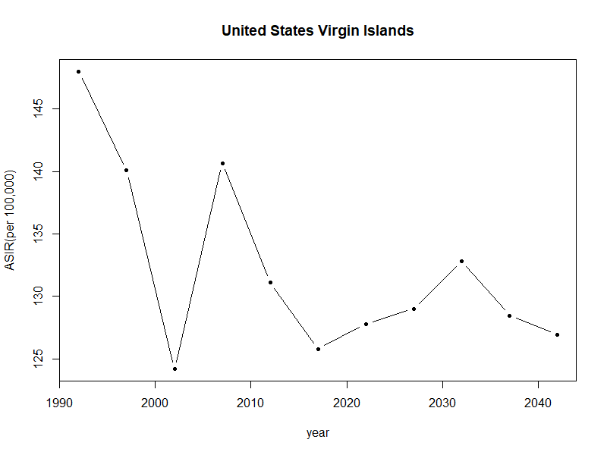


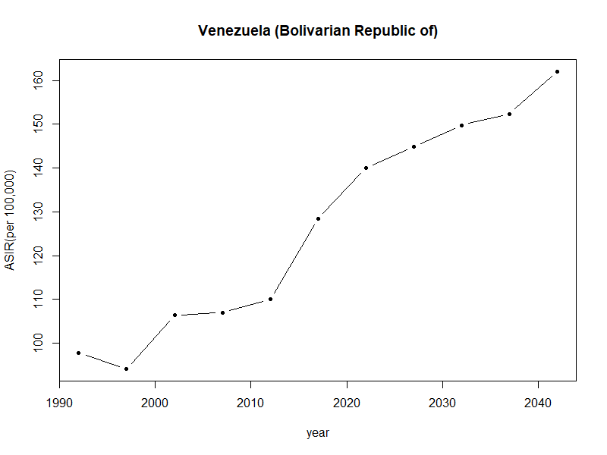


## North Africa and Middle East (21 countries)


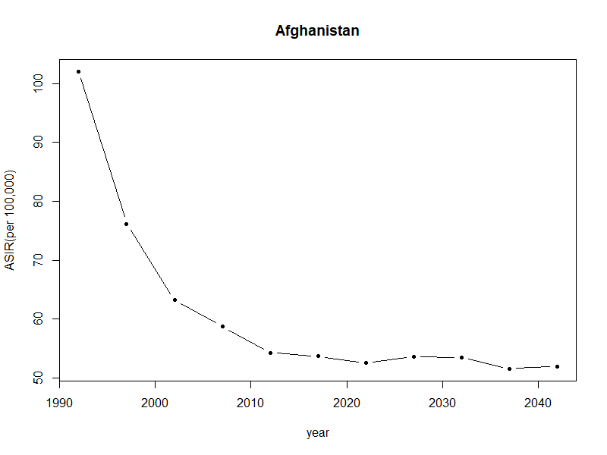


## South Asia (5 countries)

## Southeast Asia, east Asia, and Oceania (34 countries)

## Sub-Saharan Africa (46 countries)
